# From Bench to Use: The Status of Gamma-Shielding Nanomaterials and the Prospects for Lead-Free Wearables

**DOI:** 10.3390/nano15231799

**Published:** 2025-11-28

**Authors:** Qianhe Qi, Liangyu He, Hao Ye, Ce Wang, Ping Hu, Yong Liu

**Affiliations:** 1 Beijing Key Laboratory of Advanced Functional Polymer Composites, College of Material Science and Engineering, Beijing University of Chemical Technology, Beijing 100029, China; 2 Alan G. MacDiarmid Institute, Jilin University, Changchun 130012, China; 3 Department of Chemical Engineering, Tsinghua University, Beijing 100084, China

**Keywords:** γ-ray shielding, nanomaterials, high-Z fillers, polymer-based composites, multilayer structures, secondary-radiation shielding, sustainable development

## Abstract

The rapid development of deep-space exploration and crewed missions makes efficient, lightweight, and low–secondary-radiation γ-ray protection in complex cosmic fields a critical materials challenge. Current studies still struggle to simultaneously balance attenuation efficiency, areal density and thickness, flexibility, and shielding against secondary γ rays. Compared with existing reviews that mainly focus on single matrices (especially polymers) or medical lead-based protection, this work targets γ-ray shielding under deep-space and mixed radiation environments, emphasizing multiscale structural designs (multilayer/gradient architectures, micro/nanofiller synergy, and fiber networks) for suppressing secondary γ-rays and outlining composition–structure–morphology–coupled strategies for flexible, wearable, lead-free shields. Recycling and sustainability remain key bottlenecks for practical deployment. Accordingly, this review also summarizes representative Monte Carlo simulation tools and their integration with experiments, and proposes directions for element selection, structural design, and green manufacturing to build design rules and a scale-up roadmap for next-generation lead-free γ-shielding wearables.

## 1. Introduction

**Figure 1:**
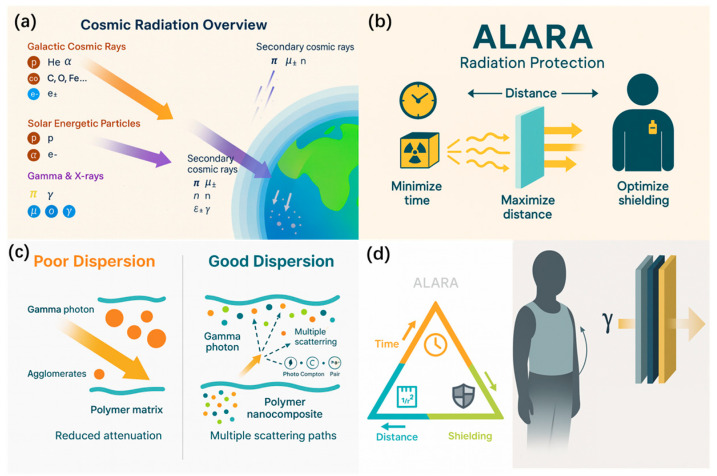
(**a**) Particles of cosmic rays; (**b**) Three elements of the ALARA principle; (**c**) γ-ray attenuation by nanofillers; (**d**) Polymer-based flexible and wearable shielding.

As human exploration activities increase, how to shield against high-energy radiation has become a pressing issue. Among the particles composing cosmic rays, γ-rays, also known as gamma particle streams, are photons that carry no electric charge and have no mass, belonging to a form of electromagnetic waves. However, their wavelength is shorter than that of X-rays, and their frequency is higher, resulting in stronger penetration power and greater energy (10 GeV–10 TeV). Very-high-energy γ-rays with energies ≥ 30 GeV are a type of ionizing radiation that can cause more severe damage to human health. Meanwhile, heavy elements with good attenuation performance for γ-radiation may also generate secondary γ-rays due to inelastic scattering or radiative capture during interaction, as shown in Figure 1a [1,2]. Therefore, the actual radiation environment is a complex mixed field composed of γ-rays, high-energy protons, heavy ions, and secondary neutrons. Prolonged human exposure to γ-radiation can cause irreversible harm to the body, seriously endangering human health and safety [3,4]. To reduce radiation damage to the human body, the International Radiation-Protection Association (IRPA) has proposed the ALARA principle based on three safety parameters, indicating that protective materials are crucial. Shielding systems should achieve a balance between attenuation efficiency and secondary-radiation suppression [5,6], as illustrated in Figure 1b.

This balance is particularly pronounced in three representative scenarios: deep-space radiation protection, medical shielding, and wearable/flexible protection. For deep-space missions, galactic cosmic rays and their secondary γ-radiation span a broad energy spectrum of approximately 0.1–100 MeV. Transport calculations indicate that when the areal density of structural shields (such as Al or hydrogen-rich polymers) is increased to about 10–20 g·cm^−2^, the equivalent dose to astronauts is already close to optimal, and further thickness leads to only marginal benefit or even degradation of protection because of the buildup of secondary neutrons and light ions. Approximating this regime with monoenergetic γ rays, design data for ^137^Cs (0.662 MeV) and ^60^Co (1.17/1.33 MeV) give typical half-value layers (HVLs) of about 0.7–1.2 cm of Pb (≈8–14 g·cm^−2^) and 4.8–6.6 cm of ordinary concrete (≈11–16 g·cm^−2^), which define the mass scale of “bulk shielding” materials. In contrast, diagnostic and interventional radiology mainly involves X/γ photons in the range of 30–150 keV, where 0.25–0.5 mm Pb-equivalent protective garments are sufficient to reduce occupational doses by roughly one order of magnitude, with areal densities of only about 0.3–0.6 g·cm^−2^. Building on this, wearable and flexible shields are typically optimized for 60–120 kVp beams or around 0.662 MeV (^137^Cs) by incorporating high-Z fillers into various matrices to achieve lightweight yet efficient attenuation. At 0.662 MeV, reported mass attenuation coefficients for such composites are typically about 0.08–0.15 cm^2^·g^−1^, corresponding to HVLs of roughly 2–4 cm and areal densities of about 3–6 g·cm^−2^; on a per-mass basis, their γ-ray attenuation capability is comparable to or even better than that of ordinary concrete, while offering much lower bending stiffness and superior garment integration. This cross-scenario quantitative comparison illustrates that deep-space structures primarily aim to suppress secondary particle production under strict areal-density constraints, medical bunkers favor thick, low-cost concrete to withstand high-energy beams, and wearable protection must deliver relatively high MAC at very low areal density while maintaining comfort and flexibility, thereby underscoring the need for lightweight composite designs [7,8,9,10].

Numerous comparative studies show that nanofillers, owing to their smaller size, larger specific surface area, tunable interfaces, and more uniform dispersion, often outperform micron particles in shielding performance and exhibit higher thermodynamic properties, as shown in Figure 1c. They also show potential in flexible and wearable shielding [11,12], as shown in Figure 1d. This approach has been repeatedly validated in systems with a variety of different matrices.

Against this background, this review focuses on lead-free or low-lead, lightweight, and often flexible γ-ray shielding systems, and is intended to provide fundamental data and design concepts for γ-ray protection to support the development of advanced integrated shielding material systems. To facilitate a clear understanding, a unified set of performance metrics is adopted: the linear attenuation coefficient (μ, LAC, in cm^−1^), mass attenuation coefficient (μ/ρ, MAC, in cm^2^·g^−1^), and half-value layer (HVL, in cm, HVL = ln2/μ) are used to quantitatively describe γ-ray attenuation. In mass-sensitive applications, HVL is also expressed in terms of areal density (ρt, in g·cm^−2^). This set of definitions establishes a consistent benchmark for comparing different material systems and structural configurations in subsequent sections, ensuring comparability across three typical application scenarios: deep-space, medical protection, and wearable shielding.

## 2. Interaction Between γ-Rays and Matter

The interactions between γ rays and materials are mainly four-fold: the photoelectric effect, the Compton effect, the pair-production effect, and coherent scattering. Understanding the interactions between γ rays and materials across different energy ranges is conducive to the better selection of absorbing materials. Among these, the first three effects make the major contribution.

**Figure 2:**
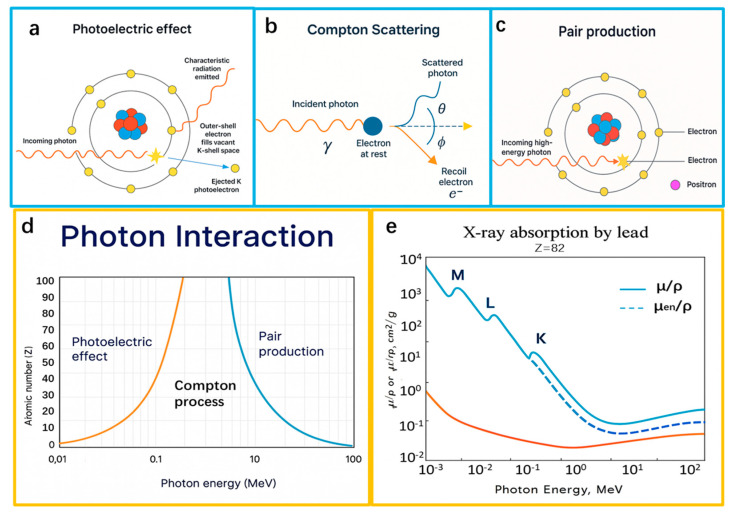
(**a**) Photoelectric effect; (**b**) Compton effect; (**c**) Pair-production effect; (**d**) Dominant photon–matter interactions across energy ranges; (**e**) Absorption-edge phenomenon. Reproduced with permission from Alsaab and Zeghib, *Polymers*; published by MDPI, 2023 [14].

First, in the low-energy region, the photoelectric effect dominates (as shown in Figure 2a). In essence, γ rays are photons with different energies; when an atom absorbs a photon, the photon undergoes elastic scattering with an extranuclear electron and transfers its energy completely to that electron, the electron breaks free of its binding to become a photoelectron, and the incident photon is completely absorbed. If the emitted electron is an inner-shell electron, the atom is left with a vacancy, and an outer-shell electron transitions inward and emits characteristic X-rays; when the incident energy is sufficiently large, Auger electrons may also be produced. The cross-section of the photoelectric effect increases with the atomic number of the absorbing atom and decreases with increasing photon energy.

When the energy of incident photons increases, the Compton effect occurs (as shown in Figure 2b). γ photons undergo inelastic scattering with outer-valence electrons, transferring part of their energy and momentum to the electrons; the photon energy decreases, its propagation direction is deflected, and the ejected electron is called a Compton electron. The Compton electrons and the scattered photons then continue to interact with the absorbing material until their energy is dissipated. The energy difference between the incident and scattered photons is approximately equal to the kinetic energy of the Compton electron. Compared with the photoelectric effect, the probability of the Compton effect depends more weakly on the atomic number (being mainly related to the electron number density), decreases with increasing incident photon energy, but at a slower rate than the photoelectric effect.

When the energy of the incident photon exceeds 1.02 MeV, the photon is converted into a positron and an electron in the electric field around the atomic nucleus (as shown in Figure 2c); the rest energy and kinetic energy of the two are derived from the energy of the incident photon. The generated positron and electron subsequently collide or otherwise interact with electrons in the absorbing medium, and so on, until their energy is completely dissipated. As with the photoelectric and Compton effects, the probability of the pair-production effect is also proportional to the atomic number [13].

In summary, γ–matter interactions are jointly determined by photon energy and the atomic number of the material. In the low-energy region (≤500 keV), the photoelectric effect dominates, and the attenuation coefficient of matter is relatively high; at this time, the photon energy is almost completely transferred to electrons, and photoelectrons are emitted, with a pronounced dependence on material composition. As the energy increases to the intermediate-energy region, Compton scattering becomes the main mechanism; this interaction mechanism does not depend on the atomic number of the elements in the material, is insensitive to elemental differences, and its probability of occurrence depends mainly on the number of electrons per unit mass, being proportional to the ratio of atomic number to atomic weight, Z/M. When the photon energy exceeds about 1.02 MeV, the dominant mechanism shifts to the pair-production effect, and the dependence on the atomic number of the material strengthens again, as shown in Figure 2d. For a clear material-to-energy mapping, the 30–200 keV range is defined as the low-energy region dominated by the photoelectric effect, 200–1000 keV as the medium-energy region governed by Compton scattering, and energies above approximately 1 MeV as the high-energy region where pair production becomes predominant.

In general, the absorption capability of materials shows an overall decreasing trend with increasing photon energy, as demonstrated in Figure 2 from Ref. [15]. The difference between the energy of the incident photon and the kinetic energy of the photoelectron is the binding energy of the electron in that shell; therefore, when the incident photon energy approaches the binding energy of a given shell, the photoelectric effect is more likely to occur, and when it exactly equals the binding energy, the probability is maximal. This phenomenon is called the absorption-edge effect, in which the collision probability increases, the absorption strength is significantly enhanced, and the absorption coefficient (μm) suddenly increases, indicating that the photon energy is completely absorbed by electrons with a high probability [16]. Consequently, the reaction cross-section increases abruptly at the K, L, and M shells of the atom [14,17], as shown in Figure 2e.

From the standpoint of fundamental physical laws, the emergence of entirely new interaction types between γ-ray and matter interactions is unlikely. However, “effective new mechanisms” can still be constructed through material structuring and interfacial engineering to achieve leap-type improvements in shielding performance. For example, introducing multiple-scattering pathways at the nanoscale, modulating electron-density distributions at heterogeneous interfaces to amplify secondary-electron contributions, or using multilayer architectures to “reshape” the incident energy spectrum can all significantly influence photon transport and energy dissipation without altering the underlying interaction mechanisms. It should be emphasized that the high specific surface area and relative low density of nanoparticles do not change the photon-interaction cross-sections of individual atoms; their advantages are mainly manifested at the structural level: at a given mass fraction, reducing the particle-size facilitates a more homogeneous and dense dispersion of the high-Z phase in polymer/rubber matrices, reduces agglomeration and voids and increases the interfacial area, thereby increasing the effective atomic number and electron density and markedly enhancing photoelectric absorption in the low-energy region that is highly sensitive to the local electronic structure; whereas in the medium- and high-energy regions, attenuation is mainly governed by the bulk average electron density, and its sensitivity to particle size and interfacial structure decreases, which weakens the differences between micro- and nanoscale systems.

Therefore, from the perspective of material selection, the primary route to improving γ-ray shielding is to increase the atomic number and density of the material. At a given areal density, increasing the density or thickness of a material can significantly increase the probability of interactions between photons and its extranuclear electrons, causing energy to dissipate more rapidly and manifesting as higher attenuation performance. Because the absorption edge of a single element is fixed, to cover a wider energy range, it is advisable to use combinations of multiple elements to achieve energy complementarity of absorption edges and thereby achieve optimal shielding performance [18].

## 3. γ-Ray Shielding Materials

The preferred elements first point to heavy elements, namely lead, bismuth, tungsten, and parts of the rare-earth metals. These elements have higher electron density and more tightly arranged electronic shells, with stronger overlap of electron clouds. As a representative of high atomic number and high density, lead was among the earliest to be investigated as a γ-ray shielding material owing to its high density, strong shielding capability, and relatively low processing cost. Early studies mostly used lead powder or lead compounds (PbO, Pb_3_O_4_, Pb(NO_3_)_2_, etc.) as the shielding phase, combined with polymer matrices (polyethylene, polyurethane, epoxy, rubber, etc.) to prepare composite systems, forming multiple mature routes from lead–rubber to lead–salt-modified resins and exhibiting good attenuation of low-energy and medium-to-high-energy photons. In terms of applications, leaded glass/transparent shielding is used in scenarios requiring visualization, and leaded rubber/textile-like materials are used for medical protective wear, both of which have been reported [19,20]. Although lead-based materials possess significant attenuation advantages, their health and environmental risks (toxicity and potential bioaccumulation) are prompting research and applications to shift toward safer alternative elements [21]. Therefore, identifying green, nontoxic, and low-cost lightweight alternatives has become a primary task at present.

Bismuth and tungsten have atomic numbers close to that of lead, and most of their compounds are stable and nontoxic; relative to lead, they are less toxic and greener, and the feasibility of W–Bi-based systems as substitutes for lead has been experimentally verified. Since X-rays are electromagnetic waves with lower energy than γ rays, He Dayong et al. first used electrospinning to prepare Bi_2_WO_6_/WO_3_/PAN composite nanofiber membranes and verified their excellent X-ray shielding performance. Using PAN nanofibers as the carrier, WO_3_ nanorods were first grown on the fiber surface, followed by the construction of a binary heterojunction structure with multicomponent synergistic absorption; its shielding performance surpassed that of lead glass and single-inorganic-component/PAN composite nanofiber membranes, while the flexibility of the composite pointed to wearability for personal protective clothing; Taking aluminum, a common structural material for spacecraft, as a reference, the PWAIO/PAN composite nanofiber membrane prepared by this group exhibits mass attenuation coefficients in the 30–80 keV medical X-ray energy range more than one order of magnitude higher than those of aluminum (by about 16–22 times); at 30 keV, its μ/ρ reaches 20.17 cm^2^·g^−1^, whereas that of aluminum is only about 1.1 cm^2^·g^−1^ [22]. Furthermore, to verify the good γ-ray shielding performance of Bi_2_WO_6_, B. M. Chandrika et al. prepared Bi_2_WO_6_ nanomaterials by a green combustion method, presenting a single-phase flake-like nanostructure. Measured under narrow-beam geometry with a γ spectrometer, its low-energy γ shielding performance was found to be comparable to that of lead and suitable for the energy ranges required in medical applications [23]. Moreover, Bi_2_WO_6_ with different morphologies (e.g., flake-like, flower-like) leads to differences in the shielding performance of composites, providing ideas and experimental bases for morphological control in material preparation [24]. To further compare the γ-ray shielding performance of tungsten–bismuth powders, B. Wang et al. used thermoplastic vulcanizates (TPV) as the matrix to prepare two systems, respectively adding a 1:1 physical mixture of elemental W/Bi powders and bismuth tungstate (Bi_2_W_3_O_12_), with filler loadings of 20–100 wt.%. Simulations using NECP-MCX and XCOM verified that the W/Bi elemental-powder system was superior to the BiWO compound system in terms of overall shielding and mechanical properties [25].

Yao Ya et al. investigated another oxide of bismuth, Bi_2_O_3_, incorporating it into ordinary concrete and comparing it with a PbO/concrete system; they found that the linear attenuation coefficient of Bi_2_O_3_/concrete was about 0.36–1.05% higher than that of PbO/concrete; when 25% Bi_2_O_3_ was added, the two differed only near the K absorption edges of lead and bismuth, while the curves in the other energy ranges were almost coincident, indicating the feasibility of using bismuth as a substitute for lead as the shielding phase. At the same time, to address the problem that cracks and pores in concrete readily affect practical effectiveness, switching to epoxy resin as the matrix and filling it with Bi_2_O_3_ can significantly mitigate defects; when the Bi_2_O_3_ content reaches 50%, its shielding capability is comparable to that of the concrete system, while reducing the adverse effects caused by pores [26].

Tungsten alloys, owing to their high density, high hardness, and high melting point, while also exhibiting corrosion resistance at room temperature to acids, alkalis, aqua regia, and air, are likewise regarded as excellent γ-ray shielding materials.

Under the same shielding target, the material thickness can be significantly reduced, with the required thickness being about two-thirds that of lead, while avoiding issues related to lead’s toxicity and recycling/processing costs; in terms of shielding capacity per unit mass, tungsten is second only to lead and can compensate for the weak absorption bands of lead and bismuth within certain energy ranges.

China’s abundant tungsten resources also provide a material basis for its industrial application. The processing bottleneck mainly stems from the difficulties and cost pressures of bulk processing and forming caused by tungsten’s high hardness and high melting point; to solve this challenge, compositing with polymer-based materials offers a feasible approach: Wang Jie et al. prepared composites using silicone as the matrix and tungsten powder as the functional phase, which achieved effective shielding while maintaining good tensile properties, with a relatively simple preparation process [27]; on this basis, they introduced nano titanium oxide and combined it with vacuum curing, further obtaining a lightweight, highly ductile, flexible shielding system with high mechanical and chemical stability [28].

To balance mechanical properties and formability, a textile-based scheme has also emerged. Xu Fujun et al. prepared shielding fabrics using tungsten monofilaments/tungsten-alloy fibers as weft yarns and chemical fibers or natural fibers as warp yarns, combining impact resistance with good shielding performance; these can be used alone or as reinforcements for composites, effectively alleviating the insufficient flexibility of traditional tungsten materials due to their high density [29].

For the surfaces of equipment and devices, plasma thermal spraying technology can in situ build tungsten or tungsten-based cermet shielding coatings; studies have shown that, under equal-thickness conditions, the shielding effect of tungsten-based coatings is superior to that of lead coatings, verifying the feasibility of tungsten substitution from the process perspective [30]. Meanwhile, tungsten carbide (WC) nanomaterials, by virtue of their high density, high hardness, and chemical resistance, have been proven to possess the feasibility of lead-free shielding [31].

As heavy metallic elements, rare-earth elements are also widely used in the field of γ-ray shielding; they have similar atomic structures, comparable ionic radii, an electronic configuration of 4fx5d0–16s2, and a characteristic valence state of +3, with some elements being +2 or +4. They are commonly used to compensate for the weak absorption regions of single heavy metals against radiation. The K-shell absorption edges of traditional shielding elements such as lead, tungsten, and bismuth are all large, and there exist weak absorption regions for γ photons with energies below the K-shell absorption edge, requiring rare-earth elements with smaller K-shell absorption edges to absorb low-energy radiation. In the lanthanide series, as the atomic number increases, the K-shell absorption edge ranges from 38.8 keV for La to 63.3 keV for Lu; the K-shell absorption edges of several metals and rare-earth elements are shown in Table 1. K-edge values are taken from standard X-ray edge tables and the NIST X-ray Mass Attenuation Coefficients database:

Gulbicim et al. studied several rare-earth hexaborides (DyB_6_, PrB_6_, YbB_6_) and found that they exhibit considerable attenuation in the energy range of 21.0 keV to 1.728 MeV; owing to their lower density, ease of transport, and because their mass attenuation coefficients are close to those of lead when E < 1.0 MeV, they are regarded as promising lightweight shielding candidates [32]. Wenjing Wei et al. introduced Gd_2_O(CO_3_)_2_·H_2_O into a Bi_2_O_3_/epoxy matrix to construct a heterostructure that realizes a division of labor across energy ranges: Bi_2_O_3_ is more suitable for medium-to-high energies, whereas the Gd component enhances low-energy absorption; the combination not only retains medium-to-high-energy shielding but also significantly improves performance in the low-energy region, and interfacial electronic rearrangement and increased electron density are considered to increase the probability of Compton interactions, thereby achieving synergistic enhancement across low, medium, and high energies [33]. Ce doping of Bi_2_WO_6_ has likewise been confirmed to have the potential to replace lead-based materials in the low-energy region (<270 keV); the shielding performance first increases and then decreases with Ce content, reaching a shielding rate of 38.66% for 105.3 keV γ rays at 5 mol% [34]. In addition, Guowei Wang et al. employed rare-earth elements as fillers to target the weak absorption band of Bi_2_WO_6_ at 40–70 keV; doping with rare-earth Er^3+^ yields a significant gain in the low-energy region, and as the doping ratio increases, the number of nanoparticles gradually increases; when the molar fraction of Er^3+^ is 12.5%, the sample morphology changes further, locally presenting interlaced nanosheets, and the shielding performance follows a trend of first increasing and then decreasing with doping, with the mass attenuation coefficient for 59.5 keV γ rays reaching 12.6 cm^2^ g^−1^, a 25% improvement over the undoped sample [35]. Yaodong Dai et al. employed a γ-irradiation-initiated graft polymerization route to prepare poly(acrylic acid) (PAA)/epoxy-based radiation-protection composites; comparing systems incorporating rare-earth metal acrylates versus lead acrylates, samarium ions coordinate with PAA to form a uniformly dispersed organic/inorganic network within the epoxy matrix, demonstrating that, owing to the absorption-edge effect, the rare-earth element samarium affords superior shielding to Pb in the low-energy region [36].

From an engineering application perspective, Pb, Bi, W, and rare-earth shielding phases also exhibit significant differences in terms of supply chain robustness, toxicity/regulation, recyclability, and cost. Lead remains one of the cheapest and most maturely supplied high-Z elements, with a well-established closed-loop industrial chain; for instance, the recycling rate for lead-acid batteries nears 99% in Europe and the US, with recycled lead constituting the majority of consumption. However, its well-defined neurotoxicity, stringent occupational exposure limits, and increasingly strict environmental regulations for waste disposal significantly restrict its use in disposable or distributed scenarios such as wearable and protective clothing [37,38,39,40]. In contrast, bismuth, with a density close to that of lead, is regarded as a “green heavy metal” due to its much lower toxicity and has partially replaced lead in glass, solder, ammunition, and pharmaceuticals. Nonetheless, bismuth is almost exclusively obtained as a byproduct of lead, copper, tin, and tungsten smelting, with very few independent mines, resulting in limited supply elasticity. Its annual mineral production and trade volume are far smaller than those of lead, typically making its price approximately an order of magnitude higher [41,42,43]. Tungsten offers higher density and superior shielding capability against high-energy γ-rays, but its mining and smelting are highly concentrated in a few countries. Both the EU and the US classify tungsten as a “Critical Raw Material/Critical Mineral,” noting its high risk of supply disruption. In recent years, its price has often ranged to several hundred USD per ton or higher, with notable volatility [44]. Rare-earth elements (particularly Gd, Ce, Nd, Tb) provide excellent neutron capture and complementary energy-range attenuation capabilities. However, their mining, beneficiation, and separation processes are complex and environmentally costly. Global production and refining are highly concentrated geographically, and prices for key oxides like Nd/Pr/Tb can range from tens of thousands USD per ton to nearly a thousand USD per kilogram, showing high sensitivity to geopolitical factors [45]. In summary, while Pb-based systems retain advantages in cost and recyclability, they are constrained by toxicity and regulatory pressures. Bi-based fillers offer a relatively benign but more expensive and supply-limited alternative. Shielding materials with high W or rare-earth content provide technically irreplaceable performance for high-energy γ or neutron-γ mixed fields, yet their application requires careful consideration of their higher, more volatile life-cycle costs and associated supply chain risks.

## 4. Research Progress in Composite Materials

Composites are usually composed of a matrix and a reinforcing phase: the reinforcing phase bears the main load and may take the form of long/short fibers or particles; the matrix is responsible for bonding, supporting, and transmitting stress, and common matrices include rubbers, resins, metals, ceramics, and graphite. Compared with a single material, composites can achieve a better balance among shielding, mechanical performance, and formability, providing a designable platform for γ-ray protection. In view of practical needs in radiation environments, practical shielding materials must not only possess good thermodynamic stability but also adapt to mixed radiation fields, while striving to be lightweight, flexible, low-toxicity, and portable. On this basis, engineering practice often composites different functional constituents with different matrices to obtain higher attenuation efficiency under given areal density/thickness constraints, while maintaining processability and service durability of the materials.

Current research focuses on micro/nanoscale reinforcing phases. Compared with micron-sized particles, nanofillers have smaller size, larger specific surface area, and more uniform dispersion in the matrix, which can significantly increase the probability of photon–electron interactions, thereby achieving superior attenuation performance at the same or even lower filler loadings; at the same time, micro/nanofillers can also improve morphological structure and tune polymer properties (e.g., elasticity, thermal resistance, and barrier properties), thereby achieving effective attenuation under lightweight and flexible conditions. Correspondingly, issues such as agglomeration arising from the high specific surface area and the consequent declines in rheological and mechanical properties should not be underestimated, for instance, Alshahri et al. observed that within the 5–15 wt.% Bi_2_O_3_ range, the tensile strength of the composite initially increased from approximately 14.8 MPa for pure LDPE to about 15.5 MPa at 10 wt.%, followed by a decrease to roughly 13.1 MPa at 15 wt.%. Concurrently, the elongation at break decreased from about 378% to approximately 335%, indicating that excessive filler loading compromises both toughness and load-bearing capacity due to deteriorated interfacial adhesion and particle agglomeration [46]. Similarly, in PMMA/Bi_2_O_3_ nanocomposites fabricated by Marshall et al., increasing the Bi_2_O_3_ content from 0 to 25–50 wt.% reduced the maximum tensile stress from about 14.2 MPa to approximately 6.5–6.8 MPa. This demonstrates that very high filler volume fractions significantly weaken the load-bearing skeleton of the matrix and amplify stress concentration induced by particle agglomeration [47]. It can be mitigated through surface modification and dispersion processes. In what follows, this paper reviews the research progress of composite shielding materials by matrix type; within each class of matrix, it discusses nano/microscale reinforcement methods, interfacial and dispersion strategies, structural design, and their comprehensive impacts on shielding, mechanics, and applications, and provides reference data.

Meanwhile, in γ-ray shielding research, simulation is an experimental approach parallel to physical testing and can be used to characterize the attenuation performance of materials. Compared with direct irradiation tests, simulations can avoid exposing researchers to radiation environments and reduce experimental costs. Common γ-radiation simulation and database tools include WinXCOM, Geant4, MCNPX, FLUKA, and Phy-X/PSD. In practical studies, experimental and simulation methods are usually combined for comparison and validation; in most cases, the simulation results are in good agreement with the measured data [5,16].

It is important to note that the computational tools referenced in this work serve distinct purposes across energy ranges and physical modeling approaches. Databases and platforms such as XCOM and Phy-X/PSD, which rely on evaluated photon-interaction cross-sections, cover photon energies from approximately 1 keV–100 GeV. They provide parameters such as μ, μ/ρ, and HVL for simple slab geometries, making them suitable for material screening and analyzing energy-dependent trends, but not for accurately replicating experimental beam and detector setups [48,49]. In contrast, general-purpose Monte Carlo transport codes like Geant4 and MCNP track photons and secondary particles through arbitrary 3D geometries, typically in the keV–GeV range, allowing selection among different electromagnetic physics models. They are therefore better suited for complex shielding scenarios involving collimators, environmental scatterers, and realistic detector response [50,51]. When discrepancies with experiments persist in such simulations, priority should be given to verifying the sample geometry and density definition, the choice of physics processes and cross-section libraries, and the adequate modeling of the detector response function, rather than attributing differences simply to inaccuracies in the material’s MAC.

### 4.1. Polymer-Based Composites

It has been more than 60 years since researchers first used polymers for radiation shielding. Polymers themselves possess a certain radiation-shielding capability and, owing to their low density, ease of molding, and strong designability, are often employed as matrices in composite shielding systems to host high-Z reinforcing phases; compared with single polymers, introducing inorganic fillers (metals or their compounds) with shielding function into the matrix can significantly enhance attenuation of γ rays while retaining advantages such as flexibility, processability, and portability of the materials [16] Using common polymers such as polyethylene (PE/LDPE/HDPE), poly(vinyl alcohol) (PVA), polystyrene, polypropylene, and natural rubber as matrices, the introduction of high-Z particles forms a soft-matrix and hard-filler structure, thereby achieving higher effective attenuation at the same thickness/areal density.

Fundamentally, the shielding capability of a polymer matrix is governed by its effective atomic number, electron density, molecular configuration, and packing arrangement. In the low-energy region (<0.2 MeV), where the photoelectric effect dominates, polymers containing higher atomic number elements (e.g., Cl, Br, F, S) or aromatic rings generally exhibit higher mass attenuation coefficients. In contrast, polymers composed solely of C/H with low effective atomic numbers, such as polyethylene, rely more on their high hydrogen atom density for synergistic neutron/γ-ray attenuation. In the 0.2–3 MeV range, where Compton scattering predominates, the mass attenuation coefficient is approximately proportional to electron density. Consequently, polymers with higher bulk density and tighter packing structures—such as those with aromatic rings or rigid backbones containing strong polar bonds—typically demonstrate a lower half-value layer (HVL). In the high-energy region (>3 MeV), where pair production becomes dominant, the contribution of high-Z elements is further amplified.

First, regarding the γ attenuation capability of polymers themselves, studies have systematically measured a variety of common polymers, covering polyoxymethylene, natural rubber, poly(ethyl acrylate), poly(p-phenylene methylene), polyamide 6, polyacrylonitrile, poly(vinylidene chloride), polyaniline, poly(ethylene terephthalate), poly(phenylene sulfide), polypyrrole, poly(tetrafluoroethylene), etc.; the results show that polyacrylonitrile, natural rubber, and poly(vinylidene chloride) exhibit higher PLA shielding coefficients within a given energy region [6,52]. O. Kilicoglu et al. used numerical tools to calculate parameters such as mass attenuation coefficient and half-value layer and compared them with experimental results: taking mask-use polymers as an example, simulations comparing UHDPE, poly(vinyl chloride), poly(ethylene terephthalate), polyamide 66 (nylon 66) indicated that poly(vinyl chloride) has the greatest potential in γ-attenuation characteristics, suggesting performance differentiation and energy-region dependence among common polymers [53]. Overall, the intrinsic shielding capabilities of polymer materials differ markedly, providing baseline data for subsequent micro/nanofiller introduction and structural design [6,52,53,54,55,56]. Table 2 shows the comparison of the differences in shielding coefficients of different polymers themselves.

#### 4.1.1. Unsaturated Polyester-Based (UPR)

Focusing on γ shielding in unsaturated polyester (UPR)/polyester systems, research first started with lead-containing systems: adding PbO micro/nanoparticles to UPR can significantly improve its attenuation efficiency [57,58]; adding different lead oxides, PbO, PbO_2_, and Pb_3_O_4_, to isophthalate-based UPR (ISO) showed that the ISO/PbO system had the best shielding performance: in the low-energy region, it was comparable to concrete, barite, and steel, and in the high-energy region, it was comparable to steel and concrete [59]. These data validate the effectiveness of UPR/high-Z particles, but also face limitations arising from material toxicity and density.

For lead-free alternatives, after adding fillers represented by Bi(NO_3_)_3_·5H_2_O, Bi_2_O_3_, and Bi_2_WO_6_, experiments and simulations consistently indicated that shielding efficiency increases with doping level, and the simulation results agree well with experimental data, proving the reliability of the simulation codes [60,61,62]; more specifically, adding Bi_2_O_3_ to UPR exhibits an advantage at low energies comparable to barite but with lower weight (especially at 60 wt.%) [63]. On the other hand, CdTe-doped polyester has also been confirmed to improve attenuation parameters; a composite with 20 wt.% filler shows shielding performance in the low-energy region (60 keV) comparable to or better than some highly filled PbO/WO_3_ polymers; near 122 keV, due to the shell absorption-edge effect of Pb and W, the PbO/WO_3_ systems are more advantageous, and the deviation between experiment and WinXCOM results is less than 6%, with good mutual verification [64].

For micro/nanoscale fillers, K. Bagheri et al. first added nanoscale clay, rather than high-Z compounds, into a UPR matrix to prepare UPR/nanoclay/PbO ternary composites; 5 wt.% nanoclay combined with 10–30 wt.% nano-PbO particles (0.1–0.2 μm) achieved effective attenuation in the low-to-mid energy region, the nano-PbO particles were uniformly dispersed in the matrix and improved the material’s fracture toughness, and the addition of nanoclay enhanced the material’s thermal resistance, thereby retaining the usability of the material’s thermal and tensile properties while balancing shielding performance [65].

**Figure 3:**
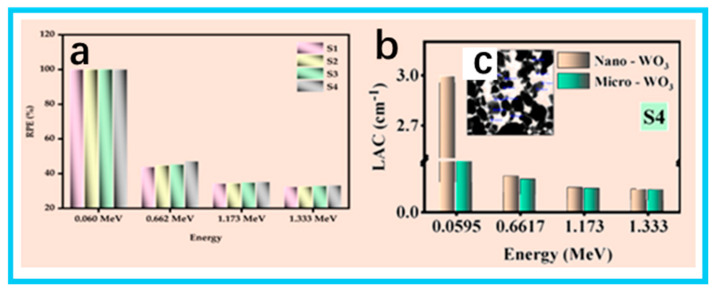
(**a**) Radiation-protection efficiency (RPE) of the prepared samples; (**b**) linear attenuation coefficient of the S4 sample with micro/nano WO_3_; (**c**) TEM image of spherical WO_3_ nanoparticles (100 nm). Reproduced with permission from Hemily et al., *Sustainability*; published by MDPI, 2022 [66].

H. M. Hemily et al. incorporated PbCO_3_ nanoparticles and varying proportions of nano-WO_3_ (≈100 nm, 5–20 wt.%) particles into a UPR/artificial marble system, first comparing the enhancement in shielding performance provided by micro- versus nanoparticles for the polymer matrix (Figure 3b). The results show that the nano-WO_3_ system achieves better shielding at the same thickness, reducing material porosity and increasing density and mechanical strength at the nanoscale; its radiation-protection efficiency (RPE) is nearly 100% at 0.0595 MeV, but decreases to about 43–46% at 0.662 MeV, indicating a pronounced energy-region dependence (Figure 3a–c) [66]. However, in a polyester/ZnO system, the combination with 50% micro- and 50% nanoparticles (20 and 30 nm) exhibits the maximum attenuation performance. Elsafi et al. attribute this to a particle-size effect—mixed particle sizes allow nanoparticles to fill the interstices between microparticles, yielding more uniform and compact packing and longer multiple-scattering paths, thereby achieving a lower half-value layer at the same volume fraction, as corroborated by experiments and Phy-X simulations [67]. In addition, simultaneously adding Mo and B nanoparticles (<100 nm) into the base unsaturated polyester resin provides a nontoxic alternative route: among the prepared formulations, the system combining 50% nano-Mo with 0% B shows the best γ shielding; Mo is proposed as one of the substitutes for lead/tungsten, while the introduction of B targets neutrons and the secondary γ rays possibly generated thereby, enabling synergistic shielding [68]. The above results are also generally consistent with studies that integrate code-based simulations (e.g., XCOM/Phy-X/Monte Carlo) [55,58,61,67,69,70]. The shielding coefficients of different filler composites in the polyester matrix are given below (Table 3) for comparison.

**Table 3:** Shielding performance of materials with different fillers added to the polyester matrix.

Polymer Matrix	Filler Type	Filler Loading (wt.%)	γ-Ray Energy E (MeV)	Mass Attenuation Coefficient μ/ρ (cm^2^·g^−1^)	Linear Attenuation Coefficient μ (cm^−1^)	HVL (cm)	TVL (cm)	Radiation-Protection Efficiency, RPE (%)
Unsaturated polyester resin (UPR) [61]	Bi_2_O_3_	50	0.662	0.1038		3.16		
UPR [62]	Bi_2_(WO_4_)_3_	20		0.0844		5.610	18.768	
UPR [64]	CdTe	20		0.0827				
UP [65]	Micro PbO	30		0.0844	0.133	5.21		
PR [66]	PbCO_3_ + WO_3_Nps	5 + 25			0.21	4.55		43–46
PR [67]	ZnO Micro + Nano	30 + 30		0.0865	0.2034	3.41	11.34	33.42
UP [68]	Mo Nps	50		0.0758		4.06		
UP [69]	Sn Nps	50			0.1516	4.5722		
UPR [70]	GNPS	1		0.08271		6.35		

#### 4.1.2. Epoxy Resin

Regarding γ-shielding studies of epoxy-based systems, early work first provided baseline attenuation parameters over a wide energy range for various resins (including epoxy), offering a reference for subsequent comparisons of their composites [71]. First, Dong Mengge et al. conducted a systematic analysis of the shielding mechanism and radiation resistance of epoxy itself in the 0.001–100 MeV energy range: the low-energy region is dominated by the photoelectric effect, the intermediate-energy region by incoherent (Compton) scattering, and the high-energy region by the combined action of incoherent scattering and pair production; attenuation increases with thickness; by comparing the macro- and micro-morphology of the resin before and after irradiation, it was found that although damage occurs under 93.5 kGy γ irradiation, the structure is not completely destroyed, indicating that epoxy itself has a certain degree of radiation resistance [72].

**Figure 4:**
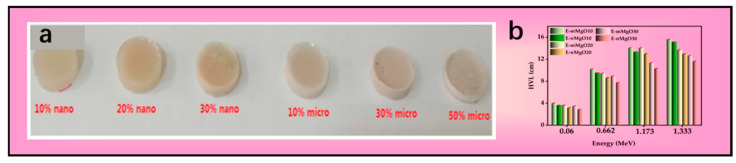
(**a**) Epoxy–MgO composites; (**b**) HVL values of epoxy-matrix samples. Reproduced with permission from Sayyed et al., *Materials*; published by MDPI, 2022 [78].

With respect to reinforcing fillers, multiple studies consistently indicate that adding high-Z or inorganic functional fillers such as Gd_2_O_3_ and WO_3_ micro/nanoparticles into epoxy increases the mass/linear attenuation coefficients with rising doping level, and that nanoparticles generally exhibit superior attenuation coefficients to microparticles (at the same or similar loadings) [73,74,75,76,77]. For example, M. I. Sayyed et al. prepared lightweight shielding composites by introducing micro/nano-MgO particles (60 ± 4 μm, 20 ± 5 nm) into epoxy; by comparing particle-size and content effects, they confirmed that increasing content and reducing particle size can both significantly enhance γ-ray shielding, with the nanoscale effect being most pronounced in the low-energy region; the experimental results agree closely with XCOM calculations (Figure 4a,b) [78]. In addition, F. Erdogan et al., using mechanical stirring, ultrasonication, and vacuum degassing followed by room-temperature curing, added 10–60 wt.% HfB_2_ nanoparticles (~60 nm) into epoxy and achieved uniform dispersion, demonstrating that with increasing HfB_2_ content, the γ attenuation coefficient of the composite increases, the HVL decreases, the neutron attenuation coefficient increases, and the microhardness rises, thereby showing its feasibility as a lightweight lead-free shielding material; on this basis, they discussed application potential in aerospace and medical packaging/protection [12]. Although increasing filler content can improve the shielding capability of the material, it may be accompanied by declines in other properties; for example, in a W-micropowder (~6 μm)/epoxy system, shielding performance increases with filler content in the 30–80 wt.% range, but particle agglomeration induces deterioration in mechanical properties, thus necessitating a balance between mechanical and shielding performance [78]. Moreover, not all nanofillers enhance the shielding performance of the matrix: for low-dimensional carbon/nitride-type functional constituents, Zhang Hongxu et al. first modified CNTs with Sm_2_O_3_/WO_3_/Bi_2_O_3_ particles and then incorporated them into the epoxy matrix; the results show that, in the presence of these functional particles, the addition of single-walled/multi-walled CNTs fails to significantly improve shielding [79].

Regarding studies on the performance of lead-free or low-lead elemental shielding materials across different energy regions, for enhancement in the low-energy region, uniformly dispersed and densely structured Er_2_O_3_/epoxy and Y_2_O_3_/GeO_2_/epoxy systems have been demonstrated to afford shielding capabilities comparable to lead under low-energy γ irradiation, demonstrating the feasibility of substitution at low energies [11,80]. Alavian et al. compared a series of Pb (~50 nm), Zn (~60 nm), Ti (~60–80 nm), ZnO (~50 nm), TiO_2_ (~20–40 nm) nanoparticle/epoxy systems: although the lead system exhibited the highest attenuation coefficient, the authors noted that the Zn and Ti systems have advantages in mechanical stability, biodegradability, and agglomeration tendency, and thus can serve as candidate materials under low-energy conditions; moreover, the attenuation parameters calculated by WinXCOM, Phy-X, and MCNPX simulations were essentially consistent with the measured results [81]. Vishnu et al. incorporated Bi_2_O_3_ and WO_3_ microparticles (<50 μm, 0–20 wt.%) into a commercial epoxy wall coating; comparisons at 662 keV showed that, to achieve the same attenuation, a thickness of 2.832 cm was required for the 20 wt.% Bi_2_O_3_ sample, whereas 4.013 cm was needed for the 20 wt.% WO_3_ sample, indicating that Bi_2_O_3_ has greater thickness efficiency at this energy; the Phy-X/PSD simulation results agreed well with the experiments, and the material exhibited desirable qualities such as flexibility, inertness, simple manufacture, and low cost [82]. In a parallel comparison of multiple filler types, WC, WC-Co, Bi_2_O_3_, BaSO_4_, and Sn/epoxy at 140 keV (thicknesses 0.7/1.4 cm) were all comparable to lead except for Sn; however, at 364 keV they were overall inferior to lead and required greater thickness to compensate, indicating that under higher-energy radiation the shielding performance of substitutes still lags behind lead [83]. To extend applicability to a wider energy range, Dong Yu et al. prepared WO_3_ and CeO_2_ nanopowder (50 nm)/epoxy multilayer composites via surface treatment; experimental results showed that the multilayer configuration with a WO_3_ layer preceding a CeO_2_ layer delivered the highest shielding performance at higher energies, because the K-shell absorption edge of W is 69.5 keV whereas that of Ce is 40.449 keV—when higher-energy photons pass through the material, W first attenuates the energy, and Ce further attenuates the reduced energy; this sequence exploits the differing magnitudes of the absorption edges, achieving complementary edges and enhanced energy-attenuation efficiency, thereby providing a strategy for doping with different reinforcing fillers [84].

#### 4.1.3. Rubber

In early studies, Gwaily et al. introduced galena (P) into NR/SBR blend rubber and confirmed that, in the intermediate-energy region, rubber-based composites can achieve significant shielding enhancement by introducing high-Z functional particles, laying the foundation for subsequent directions in flexibility and wearability [85]. Subsequently, studies on systems prepared by adding high-density barium tungstate (BaWO_4_) micropowders (<80 μm) to natural rubber (NR) and on lead-free NR/NBR/Bi_2_O_3_ systems both showed that, with increasing filler content, the mass attenuation coefficient increases, the half-value layer decreases, and the flexibility of the material allows lamination; such materials are suitable for wearable/bendable lead-free shielding scenarios (e.g., gloves, shoes, coats), demonstrating the effectiveness of replacing lead with rare high-Z oxides [86,87]. After adding Bi_3_B_5_O_12_ functional powders to natural rubber, Liang Feng found that the tensile strength and elongation of the composites were significantly superior to those of pure natural latex films, and that the modified powders were uniformly dispersed; the materials exhibited neutron–γ synergistic shielding potential [88]. Concerning particle-size effects, Asgari et al. prepared flexible rubber-based (CR/NR) composites with fillers of Pb/Bi/W in both micron- and nanoscale particles at mass fractions of 30% and 60%: within the 40–662 keV energy range, at the same loading, nanoscale fillers yield higher shielding coefficients than microscale ones, with the advantage being most pronounced in the low-energy region (dominated by the photoelectric effect); in the low-energy medical diagnostic range, Bi and Pb are more suitable filler choices [89]. For lead-free alternatives, comparative studies of micro/nanoparticles of other common functional fillers such as WO_3_ and Bi_2_O_3_ also show that both particle sizes can enhance the shielding performance of silicone–rubber-based composites, and that the combination of particle sizes and the dispersion state has an important influence on the results [90,91]. In addition to high-Z elements and their compounds, rare-earth doping has also been utilized to extend low-energy absorption capability: surface-modified La- or Ce-doped Ba_5_Ta_4_O_15_ nanopowders (100 nm) were added into natural rubber, and doping with rare-earth elements red-shifts the absorption edge; in Ta’s weak absorption energy region (59.5 keV), the rare-earth absorption edges provide compensation, further enhancing low-energy attenuation efficiency. Meanwhile, the nanosheets are homogeneously dispersed in rubber, ensuring shielding performance while also accommodating mechanical properties [18].

The incorporation of functional fillers can also improve other properties of rubber. P. Atashi et al. employed room-temperature-vulcanized PbS silicone–rubber as the matrix and prepared composites with a total of 37.5 wt.% W (<20 µm) and Bi_2_O_3_ (5 µm) microparticles as functional components, which exhibited overall shielding coefficients superior to those of a commercial 76% Pb composite in the 122–964 keV range; among them, the formulation with W/Bi_2_O_3_ = 18.75/18.75 wt.% showed (Among these formulations, the one with W/Bi_2_O_3_ ratio of 18.75/18.75 wt.% exhibited) the smallest half-value layer in the high-energy region, affording better thickness economy. Meanwhile, as the Bi_2_O_3_ fraction increased, the material’s tensile strength and elongation at break increased (correlated with reduced agglomeration). This system exhibits good γ-ray shielding and flexible processability and can serve as a lead-free alternative for flexible shielding components [92]. Table 4 below shows the differences in shielding coefficients of materials in resin and rubber matrices when different fillers are added.

#### 4.1.4. Polyolefins

Polyethylene (PE), owing to its chemical stability, light weight, and good neutron-moderating capability, has long been employed as a radiation-protection matrix; common types include low-density polyethylene (LDPE), high-density polyethylene (HDPE), and ultra-high-molecular-weight polyethylene (UHMWPE). Early studies centered on lead–boron polyethylene: HDPE systems incorporating 10 and 50 wt.% PbO in bulk and nanoparticulate forms showed competitive performance in the low-energy region comparable to barite, steel, and concrete, and were identified as suitable for flexible scenarios such as protective clothing [93,94]. Subsequent work gradually shifted to low-lead/lead-free systems: for example, adding 2 wt.% B_4_C to PE; adding 0–2 wt.% Bi_2_O_3_ nanoparticles to UHMWPE; and incorporating small proportions of nano-B_4_C (0.1–10 µm) and W nanopowders (60–80 nm)—all yielded measurable improvements in γ attenuation relative to the neat matrix [95,96,97]. In addition, Fe/Al (30 wt.%) nanoparticles and their oxide Fe_3_O_4_ (1.16–7.74 wt.%) nanoparticles, as well as ZnO nanoparticles (10–40 wt.%) in LLDPE, were confirmed by MCNPX/XCOM/XMuDat simulations and experiments to enhance shielding in the low-to-intermediate energy range; nanoscale systems exhibited higher attenuation coefficients than microscale ones. The higher the energy, the more the shielding capability depends on the content of added nanoparticles; This is attributed to the more uniform dispersion of nanoparticles in the polyethylene matrix and their larger interfacial area, which can more effectively increase the effective atomic number and electron density and thus enhance photoelectric absorption in the low-energy region that is highly sensitive to the local electronic structure; whereas in the medium- and high-energy regions, attenuation is mainly determined by the bulk average electron density and is less sensitive to particle size and interfacial structure. Moreover, the results of the three programs were in good agreement, while also emphasizing that, compared with lead, the composites are lighter and easier to process [98,99,100,101].

To achieve effective dispersion of nanoparticles rather than merely increasing the mass fraction of functional fillers, S. Kim et al. mixed functionalized W–BNNS (W nanoparticles; 10–20 nm/BNNS = 1:1; total filler 20 wt.%) into LLDPE and obtained W–BNNS/PE composites by compression molding. Microscopic observations showed W nanoparticles uniformly anchored on BNNS surfaces, suppressing BNNS restacking and avoiding W agglomeration; this configuration enlarges the effective absorption cross-section for neutrons and γ rays and improves interfacial bonding and dispersion with the polyethylene matrix [102]. To improve compatibility and dispersion of nanoparticles in polyethylene, Zhao Sheng et al. surface-modified nano-Gd_2_O_3_ (200–500 nm) with the silane coupling agent KH570, and prepared M-nanoGd_2_O_3_/B_4_C/HDPE composite shielding materials by hot pressing; SEM images indicate more uniform particle dispersion and better interfacial bonding in the HDPE matrix after modification, while mechanical and DSC tests both demonstrate performance enhancement, achieving simultaneous improvement in thermomechanical properties and combined neutron/γ shielding [103]. Kim et al. first produced PE-coated nano-W particles by ball-milling and then dispersed them into an ethylene–propylene rubber (EPM) matrix; the study showed that when the nano-W content is 15 wt.% (or 7.5 wt.% for micron-sized W), the material exhibits significant γ-attenuation enhancement in the ≥300 keV energy region, providing a strategy for structured dispersion of high-Z nanoparticles in polyolefin matrices [104].

Composites with different fillers still exhibit pronounced energy-region dependence. Taking LDPE as an example, when the polyethylene content in clay/LDPE composites is increased from 0 to 30 wt.%, EGS5/XCOM/Phy-X simulations show that the shielding capability instead decreases, reflecting that the volume fraction of inorganic fillers is the key factor governing attenuation [105]. In linear low-density polyethylene (LLDPE) with 10–30 wt.% PbO/WO_3_ powders added and tested by energy regions: the shielding performance of the composites decreases rapidly with increasing energy in the low-energy region (<500 keV); decreases only slowly in the intermediate-energy region (500–1100 keV); and changes insignificantly with energy in the high-energy region (≥1100 keV). Under high-loading conditions, the shielding coefficient of the composites can reach about 99% of that of elemental lead, and filler contents exceeding 25 wt.% are generally superior to the control data for barite and ordinary aggregates [106]. Using waste HDPE as the matrix, pressed samples containing 40–60% CuO and 5–25% phosphotungstic acid (PTA) nanoparticles exhibited, in FLUKA/MicroShield calculations over 0.015–15 MeV, a general trend of decreasing attenuation coefficient with increasing energy, and the addition of PTA could further enhance attenuation performance [107]. Targeting low-energy γ rays, CdO/HDPE systems with 10–40 wt.% micro/nanoparticles (0.58–0.95 μm, ≈50 nm) provide reference data: at 59.53 keV, the composite with 40 wt.% CdO nanoparticles achieve a mass attenuation coefficient of about 55% that of lead and 44% that of cadmium, with densities approximately 7.2 and 5.5 times lower than those of lead and cadmium, respectively; the higher thickness efficiency in the low-energy region, together with the low-density advantage, renders it an ideal lead-free alternative material [108].

**Figure 5:**
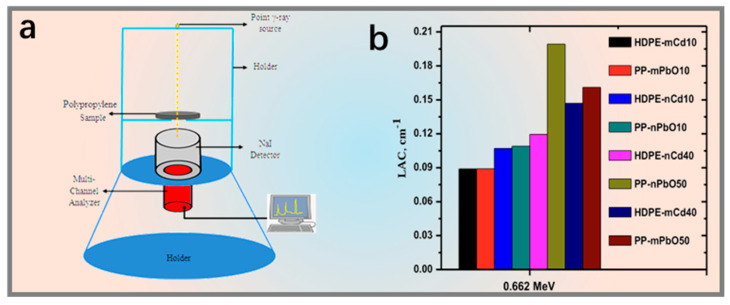
(**a**) The illustration setup of the experimental work. Reproduced with permission from El-Khatib et al., *Polymers*; published by MDPI, 2022 [109]. (**b**) Mass attenuation coefficients of different samples. Reproduced with permission from El-Khatib et al., *Materials*; published by MDPI, 2022 [110].

Polypropylene (PP) is characterized by its low density, chemical resistance, and balanced mechanical strength and processability. The team of El-Khatib incorporated micro- and nano-sized Bi_2_O_3_ (100 µm, 20 ± 5 nm; 10, 50 wt.%) and micro- and nano-sized PbO particles (10–50 wt.%) into PP to develop two types of composite shielding systems. Both micro- and nanoscale particles significantly enhanced attenuation. Measurements showed that the attenuation of the materials increased with higher filler content at commonly used energy points (including 0.662 MeV), and at the same filler content, the nano-filled systems exhibited superior shielding performance compared to the micro-filled systems. Furthermore, a comparison with HDPE filled with micro-/nano-CdO particles (0.95 µm/50 nm) revealed that the PbO/PP system outperformed the CdO/HDPE system at a photon energy of 0.662 MeV (Figure 5) [109,110].

In another study, Zakaly et al. used PP as the matrix and added nano-cadmium oxide (CdO-NPs) and nano-bentonite (Bent. NPs) to prepare ternary nanocomposites, with simulations performed using FLUKA. The results indicated that a higher CdO content led to a greater linear attenuation coefficient. Since bentonite has a lower atomic number than CdO, its contribution to shielding performance was relatively minor, indicating that the high-Z phase plays the dominant role [111].

Table 5 provides a comparison of the shielding performance of polyolefin matrices with different fillers.

#### 4.1.5. Vinyl Polymers

As an extensively utilized thermoplastic, poly(vinyl chloride) (PVC) features low cost and offers multiple forming methods. Through plasticization or crosslinking, it can be tuned between rigidity and flexibility; under flexible formulations, it can still maintain high elongation at break, and tubes, sheets, films, and complex components can be fabricated. Kassem et al. added 1–6 wt.% nano-BiVO_4_ powder (30.68 nm) into PVC by a solution-casting method to construct lightweight, flexible, lead-free shielding films. The BiVO_4_ nanoparticles are relatively uniformly dispersed in PVC with slight agglomeration. MCNP5 simulations and comparison with Phy-X/PSD theoretical values show that the composite’s linear attenuation coefficient increases with filler loading; compared with various reference shielding materials, the HVL of PVC/6% BiVO_4_ at typical energies is lower than that of some epoxy/Bi_2_O_3_, HDPE/PbO, and concrete samples [112]. Toward a sustainable pathway, Nasrabadi et al. prepared micro/nano MgO/PVC composites (1 μm/100 nm) with different loadings and evaluated their shielding performance in the 0.05–1.332 MeV range using the MCNPX simulation program: compared with microparticles, the nanosystems improve the mass attenuation coefficient to a greater extent, with enhancements of 0.84–9.97% at 25 wt.%, 7.52–12.79% at 30 wt.%, and 4.43–23.48% at 50 wt.%; moreover, in the high-energy region of 1.173–1.332 MeV, a 50 wt.% nano-MgO/PVC exhibits shielding capability that can surpass lead, and when used for protective clothing, it yields an approximate 36.46–11.13% weight reduction relative to lead, demonstrating lead-free lightweight potential [113]. El-Sharkawy et al. embedded 0–35 wt.% Bi_2_O_3_ nanoparticles into recycled PVC (r-PVC) sheets; as the loading increased, shielding improved, with the material’s half-value layer (HVL) decreasing from 11.974 cm (5 wt.%) to 7.869 cm (35 wt.%), verifying the feasibility of a lead-free plus plastic-waste upcycling route [114].

Poly(vinyl alcohol) (PVA) is obtained by hydrolysis of poly(vinyl acetate), and its water solubility and excellent film-forming ability make it a common choice as a flexible radiation-shielding matrix. The addition of Bi_2_O_3_, Pb(NO_3_)_2_, and micro/nano WO_3_ particles has been demonstrated to significantly improve the shielding performance of PVA composites, and a pronounced particle-size effect establishes the necessity of optimizing particle size [115,116,117,118]. Srinivasan et al. compared shielding performance at different filler contents in a PVA/iron-oxide system and found that adding about 0.5% magnetite achieves the best shielding performance, demonstrating the enhancement effect of small-dosage functional fillers on shielding [119]; meanwhile, tests of PVA–BaTiO_3_ nanofilms (50–56 nm) in the 81–444 keV energy range show that the mass attenuation coefficient decreases with increasing energy, and within a loading range of 0.65–3.57 wt.% a balance can be achieved among shielding, film formation, and material properties, striving to preserve filmability and flexibility while enhancing shielding, thereby providing a strategy for preparing lightweight polymer composites [120].

Poly(methyl methacrylate) (PMMA), as a transparent film-type polymer, has been widely studied for shielding; first, Manjunatha et al. measured, in the 0.015–15 MeV energy range, the linear and mass attenuation coefficients, effective atomic number, and electron density of PMMA and Kapton polyimide, two materials commonly used in aerospace, and calculated the energy absorption buildup factor (EABF) and exposure buildup factor (EBF) over a wide energy range using a geometric progression fitting method, providing important data for the γ-shielding field [121]; incorporating nano-SiO_2_ (~12–15 nm; 0.5 wt.%) and ZnO (~260–400 nm; 0.5–2 wt.%) particles, CMT (Ca_2_B_6_O_11_·5H_2_O) microparticles (5–10 μm), and WC micro/nanoparticles (20–60 wt.%) into PMMA films has been demonstrated to enhance shielding performance, reflecting the possibility of lead-free alternatives [122,123,124]. Using Bi–PMMA (0.08–1 µm; 40–60 wt.% Bi micro/nanoparticles) as an example, at 59.5 keV, the system with 60 wt.% Bi can achieve a shielding coefficient comparable to lead while offering comprehensive advantages such as ease of processing, lower weight, flexibility, and low toxicity [125]; ternary composites prepared by A. M. Ismail et al. with BaTiO_3_ nanoparticles (~39.9 nm) can further improve shielding performance, but film transparency decreases with increasing filler content, and a doping level of 10 wt.% has been shown to achieve a better balance between shielding and transmittance [126].

#### 4.1.6. Other High-Performance Polymer-Based

Polyimide (PI), owing to its heat resistance and chemical stability, is extensively employed as a shielding material that operates long-term under high temperature and strong irradiation. PI binary systems with micro/nano Bi_2_O_3_ particles, as well as PI ternary systems incorporating nano h-BN (3, 7, 11 wt.%) and Gd_2_O_3_ (1, 2, 3 wt.%) particles, have been shown to enhance attenuation performance, with shielding increasing with nanofiller content and sample thickness [127,128]. In addition, researchers have introduced SiO_2_ nanoparticles (0, 0.015, 0.030, 0.045 wt.%) into a blended system of carboxymethyl cellulose (HVCMC), poly(N-vinylpyrrolidone), and polyethylene glycol; and SeO_2_ or BN nanoparticles into a polystyrene-b-poly(ethylene glycol) (PS-b-PEG) block copolymer. Meanwhile, the flexible deformation and surface adhesion of the polymers were studied, indicating potential suitability for wearable or coating-type protection scenarios [129,130,131].

For the PVE/Pb_2_O_3_ system, when 50 wt.% Pb_2_O_3_ nanoparticles were added, the attenuation coefficient increased by about 86% compared with unfilled poly(vinyl ester)-based PVE, and the composite’s weight was approximately 17% that of lead and comparable to concrete (but heavier than polyethylene), indicating the feasibility of lightweight shielding [132].

**Figure 6:**
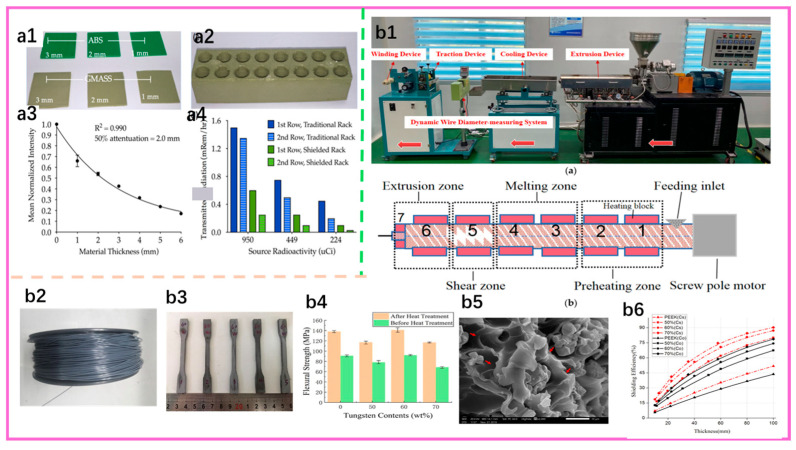
(**a1**) ABS and GMASS sheet samples (thickness 1–3 mm); (**a2**) GMASS 3D-printed test-tube rack; (**a3**) thickness–transmitted-intensity relationship for GMASS; (**a4**) comparison of transmitted radiation for shielding versus conventional racks. Reproduced with permission from Ceh et al., *Sensors*; published by MDPI, 2017 [133]. (**b1**) Laboratory setup for 3D-printing shielding filaments; (**b2**) PEEK/W shielding material printed by FDM; (**b3**) specimens for mechanical testing; (**b4**) flexural strength of PEEK and PEEK/W composites; (**b5**) SEM micrograph of the fracture surface of the 70 wt.% PEEK/W composite (×2000); (**b6**) γ-ray shielding performance of PEEK composites with different thicknesses and W contents. Reproduced with permission from Wu et al., *Materials*; published by MDPI, 2020 [134].

To meet processing needs for complex geometries, J. Ceh et al. introduced different amounts of bismuth into ABS to produce a series of 3D-printable ABS–Bi composite filaments (density ~1.2–2.7 g·cm^−3^) (Figure 6(a1,a2)), and fabricated shielding components using GMASS filaments with high bismuth content. For GMASS coupons under a ^99m^Tc γ source, the transmitted intensity decayed exponentially with thickness (Figure 6(a3)), yielding a half-value layer of about 2.0 mm; a GMASS-printed test-tube rack shielded 60–85% more γ radiation than a conventional rack, and placing the radiation source on the second row further reduced it by an additional 60–70% (Figure 6(a4)), demonstrating potential in medical imaging and shielding equipment [133].

For poly(ether ether ketone) (PEEK), which has a high melting point and is difficult to extrude, Wu et al. likewise employed three-dimensional printing (Figure 6(b1)) to prepare PEEK/W (50, 60, 70 wt.%) composites (Figure 6(b2)). Mechanical testing showed that tensile and flexural strength first increased and then decreased with increasing W content, reaching maxima at 60 wt.% (Figure 6(b3,b4)); observation of the microstructure after tensile fracture of the 70 wt.% PEEK/W composite revealed tungsten agglomeration and pronounced voids at the fracture (Figure 6(b5)). Under ^137^Cs and ^60^Co sources, γ-shielding tests showed that, with increasing tungsten content, the energy of the incident radiation decreases and the material’s shielding performance improves (Figure 6(b6)), but at the expense of heat deflection and part of the strength. Therefore, a more balanced, comprehensive performance can be achieved through heat treatment and structural optimization [134].

Beyond conventional polymer matrices, Sobczak and co-workers have recently introduced a distinct family of paraffin-based γ-/X-ray shielding micro- and nanocomposites that can be regarded as a special subclass of polymer-like shielding materials. In their earliest study, moldable paraffin–iron nano- and microcomposites (22–63 μm) were fabricated by dispersing Fe particles with sizes from tens of nanometers up to ~60 μm; the warmth-of-hands-derived plasticity enabled room-temperature reshaping, while significant attenuation of γ- and X-radiation was achieved, with HVL at low tube voltages decreasing from several centimeters for neat paraffin to the sub-centimeter range at high Fe loadings, even outperforming elemental Al in the X-ray region [135]. This concept was then extended to ternary paraffin–Fe@MWCNT systems, where 10–20 wt.% iron-encapsulated multi-walled carbon nanotube arrays not only further enhanced γ-/X-ray shielding efficiency with increasing filler content, but also imparted excellent corrosion resistance in 1 M NaCl, NaOH, and HCl solutions, while retaining room-temperature shapeability and a very simple formulation [136]. More recently, a high-efficient, manually shapeable paraffin–W microcomposite (2 μm) was proposed as a nontoxic shield, in which tungsten microparticles at different mass fractions provided strong attenuation of both γ-rays and diagnostic X-rays; in addition to radiological performance, rheological, thermal, dielectric and lubrication properties were systematically characterized, and recycling protocols were established that allow separate recovery of the paraffin matrix and W powder [137]. Complementarily, paraffin-based composites filled with dense Pb and Bi particles (10 wt.%) and with their oxides Bi_2_O_3_ and Pb_3_O_4_ (10 and 50 wt.%) were shown to be susceptible to shape change under mild heating and hand pressure, with HVL under a ^60^Co source reduced from 13–14 cm at 10 wt.% to 9 cm at 50 wt.%, while still being manufactured from only two commercially available components via a simple melt-processing route [138]. Overall, this paraffin-based family highlights an emerging route to low-melting, manually formable, and potentially recyclable shielding composites that bridge the gap between traditional solid polymer matrices and fully remeltable soft shields, offering particular promise for personalized, form-fitting protective elements and complex-shaped γ-/X-ray barriers.

It is worth noting that, for flexible/wearable shielding applications, bending, scratching, or microcracking may alter the effective thickness and the continuity of the filler network, thereby leading to changes in HVL; considering that current self-healing and shape-memory polymers generally exhibit 70–95% mechanical healing efficiency and over 90% cyclic deformation recovery [139,140], embedding these functionalities into high-Z-filler composite systems is expected to autonomously restore geometric morphology and filler-network continuity after minor damage, maintain an approximately constant HVL and attenuation performance throughout the service period, provide a quantitative design pathway for addressing bending-induced damage and fatigue cracking in next-generation flexible and wearable γ-ray shielding materials, and constitute a promising design direction for future polymer-based composite shielding systems.

### 4.2. Metal-Based Composites

Metal-based composites are usually composed of a metallic matrix and a reinforcing phase (metallic or nonmetallic); alloys are composed of two or more elements, and by adjusting composition and structure, it is possible to obtain superior hardness, tensile strength, and corrosion resistance. The crystals of metals and alloys are mostly densely packed; introducing heavy atoms/heavy-metal oxides (HMO) can further increase electron density and compactness, thereby forming a thinner γ-shielding layer for the same protection target. Aluminum- and iron-based systems, which are widely used industrially, combine advantages such as high-temperature resistance, irradiation resistance, and high thermal conductivity; taking Fe/B systems (high-boron steels, boron-containing stainless steels) as an example, owing to the low solubility of boron in iron-based matrices, Fe_2_B (high hardness, low melting point) readily forms, thus improving toughness is a key focus for subsequent optimization. In industrial practice, alloying (e.g., introducing Ti) is commonly used to improve plasticity and toughness; meanwhile, boron carbide (B_4_C), due to its high thermal stability, high hardness, and corrosion and wear resistance, has been widely used as a reinforcing filler for metal matrices [141].

First, regarding the shielding performance of industrial alloys themselves, in the 0.015–15 MeV energy range, copper–nickel (Cu–Ni) exhibits superior γ attenuation due to its higher density [142]; Akman et al. used the WinXCOM and MCNPX simulation programs for tests in the 81–1333 keV energy range, finding that Ag-enriched Ag_92_._5_/Cu_7_._5_ has a mass attenuation coefficient at 81 keV that can exceed that of lead, followed by Pd_94_/Cr_6_, Ag_72_/Cu_28_, and Pd_60_/Cu_40_; as energy increases, the gap between these alloys and lead first widens and then narrows, converging to about 10% at the high-energy end, and overall they are superior to the comparison data for brass, bronze, steel, and Al–Si alloys [5]. V. P. Singh et al. combined experimental data with WinXCOM and MCNPX simulation codes, selecting four Pd/Ag alloys; Ag_70_/Pd_30_ shows the highest mass attenuation coefficient, whereas Pd_75_/Ag_25_ exhibits the highest radiation-protection efficiency (RPE), which the authors attribute to the higher sample density—again corroborating the direct linkage between density and attenuation [143]. E. I. Majeed et al. prepared a ternary Al–Cu–PbO alloy; with PbO incorporation, Pb replaces part of Cu. The alloy’s shielding performance indicates that as PbO content increases, the alloy density increases, and shielding performance in the 0.662–1.33 MeV range rises in tandem, demonstrating the quantitative feasibility of a light-matrix/high-Z-oxide route [144].

Regarding reinforcing fillers for metallic matrices, studies on aluminum-based/B_4_C (Al/B_4_C) systems have found that when the content of micron-sized B_4_C fillers increases from 5 to 20 wt.%, the mass attenuation coefficient of the composites increases with loading; at the same loading, smaller B_4_C particle size further enhances attenuation performance, while also improving material strength. Because B_4_C possesses both neutron-moderation and neutron-capture functions, it can be used for synergistic shielding in mixed radiation fields [141]. For coating-type structures, WC–12Co flame-sprayed coatings indicate that when the energy is less than 0.05 MeV, a 0.5 mm-thick coating can achieve complete shielding; when the energy equals 0.1 MeV, 1 mm is required; whereas at 3 MeV, a 1 mm-thick coating shields only about 7.2%. In the low-energy region, the attenuation of WC–12Co coatings is superior to that of B_4_C and steel-plate controls, while B_4_C is more suitable for thermal-neutron surface shielding; this suggests that, in industrial applications, the two can be functionally complementary and arranged in graded layers [145].

The structure and configuration of alloys directly affect the effective attenuation per unit thickness. Taking foam–metal–matrix composites as an example, increasing porosity leads to higher γ transmittance, indicating that the metallic matrix bears the main attenuation role. Among combinations of various foam matrices and eight classes of fillers, MCNP-optimized results identify a tungsten-foam matrix filled with B_4_C as the optimal combination; relative to traditional Fe/water mixed shielding, it can achieve about 84.45% thickness reduction and about 47.53% weight reduction, providing a basis for lightweight protection supported by both experimental and simulation data [146]. In mixed fields, Gd_2_O_3_/W powder core–shell structures (W shell coating a Gd_2_O_3_ core) were prepared, and then co-powder-metallurgized with Al alloys and rolled into plates; these can simultaneously absorb environmental γ rays and neutrons, as well as secondary γ rays generated when rare-earth elements absorb neutrons, thereby achieving synergistic protection [147].

### 4.3. Concrete-Based Composites

Cement-based composites, owing to readily available raw materials, low cost, and controllable thickness and composition, have long been used for shielding against ionizing radiation; ordinary and heavyweight concretes, by introducing heavy aggregates (e.g., Fe_3_O_4_, BaSO_4_, etc.), increase density and effective atomic number to achieve effective attenuation over a wide energy range, while still maintaining engineering applicability under high-temperature loads and real-time service conditions—this has been repeatedly demonstrated in barite/magnetite systems and their comparative studies [148,149]. However, heating and irradiation can induce loss of chemically bound water in concrete, microcrack propagation, and increased porosity, thereby weakening its shielding and mechanical performance; to address this deficiency, micro/nanofillers, owing to their large specific surface area and high activity, can act as heterogeneous nuclei for hydration, promoting the formation of hydration products and densification of the skeleton, thus improving microstructural stability while enhancing attenuation performance [150,151]. Experiments on adding PbO micro/nanoparticles to ordinary concrete are consistent with MCNP4C simulations: at a low energy of 511 keV, an addition of only 1 wt.% can increase the mass attenuation coefficient by about two-fold, and at 5 wt.% by about three-fold; the corresponding half-value layer (HVL) decreases by about 64% and 48% at 511 keV and 1332 keV, respectively; when the dosage exceeds 10 wt.%, a “half-saturation” phenomenon appears, and the particle-size effect can be approximately neglected in this system [152].

TiO_2_ can enhance the compressive strength and wear resistance of concrete; therefore, Nikbin et al. added different amounts of nano-TiO_2_ particles to Type II ordinary Portland cement containing various oxides (SiO_2_, Al_2_O_3_, Fe_2_O_3_) and compared them with lead nanoparticles, finding that at 662 keV, adding 8% nano-TiO_2_ particles increased the concrete shielding coefficient by 9%, whereas adding 10% nano-PbO_2_ particles increased the concrete coefficient by 4.1%. The concrete shielding coefficient increases with filler content because the number of particles serving as shielding increases, and the filling characteristics of nanoparticles reduce sample porosity, thereby increasing sample density [149]. Similarly, S. Dezhampanah et al. found in a Type II Portland cement–polypropylene fiber composite system prepared with magnetite aggregate that the addition of n-TiO_2_ nanoparticles further improved the synergy between shielding and load-bearing, providing a research approach in which fiber toughening and nano-densified structures proceed in parallel [153]. S. H. Al-Tersawy et al. simultaneously introduced multi-nanocomponents of SiO_2_/Fe_2_O_3_/TiO_2_ into normal-weight concrete, maintaining lower porosity and stable shielding performance under long-term irradiation conditions [150].

For other nanoscale heavy-aggregate functional fillers, researchers added different amounts of micro/nano WO_3_ particles, Bi_2_O_3_ particles, and MnFe_2_O_4_ and SiO_2_ nanoparticles into concrete matrices, with XCOM/Geant4 simulations and experiments mutually corroborating one another: the results show that over all measured energy ranges, the linear attenuation coefficient of MnFe_2_O_4_ composites is better than that of SiO_2_ materials, Bi_2_O_3_ doping is superior to WO_3_, and nanoparticles outperform microparticles [154,155,156,157]. In OPC pastes containing Fe_2_O_3_ (0.5–3 wt.%) and ZnO (0.05–0.2 wt.%), S. A. Abo-El-Enein et al. found that the concrete linear attenuation coefficient increases significantly with hydration time; the sample doped with 2 wt.% NF has the best shielding performance, because NF can increase compactness during nucleation, thereby increasing sample density and in turn improving concrete strength, heat resistance, and the γ attenuation coefficient [151]. In a WOPC/Fe_3_O_4_ nanosystem, as the Fe_3_O_4_ nanoparticle loading increases, the sample’s attenuation coefficient indeed increases. However, when the loading exceeds 10 wt.%, adding particles does not significantly improve shielding performance, and the uneven dispersion of Fe_3_O_4_ nanoparticles in cement adversely affects microstructure and mechanical properties, indicating that heavier aggregate fails to necessarily yield better concrete performance, and that an optimal filler content should be found between shielding performance and microstructure [158].

Regarding the research approach of adding multiple nanofillers for synergistic γ-ray shielding, Nikbin et al. added nano-WO_3_ and Bi_2_O_3_ particles and their combination into magnetite heavyweight concrete; when the total loading of the WO_3_ + Bi_2_O_3_ combined nanoparticles was 6 wt.%, the composite showed the best shielding performance, with the LAC at 662/1173/1332 keV increasing by 6.57%/3.54%/4.92%, while both compressive strength and ultrasonic pulse velocity increased, and systematic values of HVL/TVL/MFP versus energy were provided [159]. Mesbahi et al., using ordinary concrete as the matrix, simultaneously added four micro/nanoparticles (PbO_2_, Fe_2_O_3_, WO_3_, H_4_B) and analyzed them with the Monte Carlo MCNPX code, finding that the average difference in γ-ray attenuation coefficients between microparticles and nanoparticles was 7.6% [160].

For barite-based ternary polymer composites adjacent to cement systems, Demir et al. added polypropylene fibers to three concretes—ordinary concrete (NC), barite concrete (BC), and siderite concrete (SC)—to prepare composites, finding that barite concrete exhibits better shielding performance at both room and elevated temperatures [124]. Gürel et al. prepared bismuth-enhanced barite-based ternary polymer composites to replace traditional lead materials. Experimental data, WinXCOM, and GEANT4 all yielded consistent conclusions: the 50 wt.% Bi sample exhibited superior low-energy shielding performance than comparable WO_3_/PMMA and nano-PbO/polymer systems, indicating that the low-energy coupling advantage of BaSO_4_ and Bi can provide new ideas for cement systems [161]. Chen Zhenfu used lead–zinc tailing sand to partially replace (0–100%) ordinary sand as fine aggregate to prepare concrete. As the replacement rate increased, the γ absorption coefficient increased, and HVL/TVL decreased, while compressive strength gradually declined; considering both mechanical and shielding performance, a doping range of 40–50% is a better replacement interval, and shielding capability increases with specimen thickness [162].

### 4.4. Glass-Based Composites

Unlike polymers or metals, glass is amorphous with disordered atomic arrangements, providing numerous sites for atomic interactions at the microstructural level. Its transparency also enables it to serve a dual role of shielding and observation in radiation environments where visual inspection is required. Existing studies cover multiple glass matrices such as silicates, borates, phosphates, and tellurites, among which tellurite glasses have attracted attention due to their low melting point, relatively high thermal stability, and large effective atomic number; glass-ceramics include systems such as aluminosilicates, borates, and borotellurites. The overall research approach remains to introduce heavy-metal oxides to increase the density and effective atomic number of glass; common fillers include micro/nanoparticles of PbO, Bi_2_O_3_, WO_3_, and MoO [163,164,165].

Focusing on the two high-Z elements Bi and Pb, numerous studies have been conducted. Mohansingh Heerasingh et al. doped lead tellurovanadate glass with CoO as a dopant while fixing the lead content; as the CoO concentration increased, the density and thermal stability of the glass samples increased synchronously, and the shielding performance also improved, indicating that the shielding performance of glass depends on its density. The sample with the highest CoO concentration exhibited shielding superior to some commercial glasses. This is the first thorough study of the shielding performance of CoO-doped lead tellurovanadate glass and provides detailed data for the development of low-lead or lead-free glass materials [163]. Sayyed et al. substituted B_2_O_3_ with Bi_2_O_3_ in three oxide matrices containing BaO, ZnO, and Li_2_O; because Bi_2_O_3_ is heavier, the substituted glass systems had higher density and therefore stronger measured shielding performance. Compared with pure lead, in the high-energy region, this type of glass can reach 65% of lead’s shielding effectiveness [166]. Considering material recycling, Sayyed subsequently added 10–30 wt.% Bi_2_O_3_ micro/nanoparticles into laboratory waste glass; the results likewise showed photon-shielding capability, with the nanoscale system accompanied by higher electron-cloud density and a larger Compton-scattering cross-section, pointing toward future low-lead or even lead-free materials [167,168]. Accordingly, Marzuki et al. systematically compared the substitution effect of Bi^3+^ for Pb^2+^ and found that replacing Bi_2_O_3_ with PbO actually reduced the linear attenuation coefficient, indicating that Bi^3+^ contributes more to γ shielding in this glass system [164]. Increasing Bi_2_O_3_ content leads to synchronous increases in density and mass attenuation coefficient; comparison between simulations and experiments shows that, except in the 0.04–0.08 MeV interval, the sample containing 40 mol% Bi_2_O_3_ outperforms commercial glasses such as RS-360 and RS-253 overall [169]. In a BaO–TeO_2_ glass system, four different oxides—Bi_2_O_3_, MnO_2_, TiO_2_, and MoO_3_—were added separately; the Bi-doped sample exhibited the highest shielding coefficient, likewise attributed to the higher atomic number of Bi leading to greater sample density [170]. In quaternary Bi_2_O_3_–(75–x)TeO_2_–20Li_2_O–5Al_2_O_3_ glasses, as the Bi_2_O_3_ content increased from 0–5 mol%, both density and mass attenuation coefficient increased; at 662 keV, the 5 mol% sample showed improvements of about 33% and 20% over BA-type concrete and lead-based phosphate glass, respectively, and at specific energies the half-value layer can be lower than those of RS253-G18 and RS360 [171].

Beyond Bi- and Pb-oxide functionality, the enhancement of glass-system shielding performance and property improvements by other functional oxides and nanoscale effects have also been widely studied. The introduction of TiO_2_ nanoparticles, NiO nanoparticles, Er^3+^, and Ba has all been confirmed to improve shielding performance, with a pronounced influence on material thickness in the low-energy region [172,173,174,175]. In glass systems co-introducing Bi and Ba, tests using XCOM and Geant4 simulations showed that the shielding performance of Bi is better than that of Ba, with more obvious improvements in the low-energy region [176]. Introducing 0–1 mol% ZrO_2_ into a Bi_2_O_3_–B_2_O_3_–MnO_2_ system had little effect on shielding, possibly because the doping level was too low; the results likewise indicate that Bi plays the dominant role in shielding within this glass system. Compared with lead-enhanced epoxy, heavyweight magnetite concrete, and certain tellurite and phosphate glasses, this sample still shows a good level of performance [177]. Selim et al. used the Phy-X/PSD program to simulate the shielding performance of Dy_2_O_3_-doped borosilicate glass in the 662–1333 keV range; the system exhibits a non-monotonic variation of the minimum half-value layer with Dy content, attaining a relative minimum at 2 mol%, and in the same energy range it can outperform ordinary concrete, several iron-ore concretes, and some borate and neodymium glasses [178]. Adding WO_3_ nanoparticles to TeO_2_–B_2_O_3_–ZnO glass, FLUKA simulations showed that the undoped sample had the best shielding parameters, but the introduction of WO_3_ increased the bandgap and reduced the refractive index, so a balance between shielding and optical properties must be considered [179]. Incorporating BaTiO_3_ into BaO–B_2_O_3_–MgO–Na_2_O–CaO glass can increase both density and shielding performance, but the MBT2.5 glass with 2.5% BaTiO_3_ had the highest mechanical hardness, and further addition led to decreased hardness; thus, a good balance between mechanical and shielding performance is needed [180]. In Na_2_Si_3_O_7_ glass, introducing micro- and nano-Ag particles can enhance low-energy shielding, though the difference between the two particle sizes is not obvious [181]. When bulk and nano Bi_2_O_3_ were introduced into glass, the mass attenuation coefficient at the low-energy end of 0.5953 MeV increased significantly with content; by the high-energy end of 1.41 MeV, the improvement diminished, and nanoparticles showed better dispersibility [182]. J. A. Elias et al. incorporated 1 or 5 wt.% nanoparticles of Mn, Cu, and Cr into Li_2_B_4_O_7_ glass, and Phy-X/PSD simulations indicate that, in the 0.03–1 MeV range, Cu-doped samples provide the best shielding, followed by Mn and then Cr [183].

Table 6 below presents the radiation-shielding coefficient values of different glass systems.

### 4.5. Ceramic-Based Composites

Ceramics are inorganic, nonmetallic, polycrystalline, multiphase materials with high-temperature stability, corrosion resistance, and good insulation, and they also exhibit a certain attenuation capability over a relatively wide energy range; when powdered PbO is used as a dopant, and the PbO content is increased from 2.5 wt.% to 10 wt.%, SEM microstructure images first show that, regardless of the PbO dosage, it aggregates uniformly in flake-like clusters, is anchored between clay crystals, and is evenly distributed; at 59.53 keV the mass attenuation coefficient of the samples increases from about 0.20112 cm^2^ g^−1^ to about 0.29317 cm^2^ g^−1^; with increasing energy, the attenuation coefficient shows a decreasing trend, and the experiments agree well with XCOM simulation calculations [184]. When nano-PbO particles are used instead as dopants in the same type of ceramic system, studies show that, with increasing nano-PbO content, the shielding performance in the same energy region increases synchronously, indicating that nanoscale doping, provided dispersion is ensured, yields a more effective improvement in attenuation performance [185]. Taking mineral-based ceramics as an example, Asal et al. prepared and tested bentonite-based ceramics and systematically provided the linear/mass attenuation coefficients, mean free path, and half-value layer at different energies; the results show that increases in sample thickness and density simultaneously reduce HVL/TVL and enhance attenuation capability, with experimental values in good agreement with database/model-based calculations; meanwhile, the filler (bentonite/additive ratio) and sintering method directly alter the ceramic microstructure (porosity, pore size, and degree of grain bonding), and more densified ceramics exhibit lower porosity and higher bulk density; therefore, samples that are denser and less porous have higher attenuation coefficients and lower HVL [186]. Overall, combining matrix densification of ceramics with the introduction of high-Z oxides likewise achieves good shielding performance in the low-to-medium-energy regions. Compared with other matrix composites, literature on ceramic-based γ shielding is noticeably less abundant due to limitations such as brittleness and low allowable deformation. However, owing to their heat resistance, corrosion resistance, insulation, and stable dimensions and properties, ceramics are more suitable for liners of shielding equipment housings, fixed shielding components around nuclear facilities or accelerators, and wall claddings (such as tiles/ceramic plates containing heavy-metal oxides like PbO and Bi_2_O_3_), as well as for serving as functional phases in matrices such as concrete and glass to participate in synergistic shielding.

## 5. Conclusions

This review addresses the critical need for γ-ray shielding in deep-space and complex radiation environments, systematically examining the progress in using nano/micro-sized high-Z fillers within various matrices, including polymers, metals, concrete, glass, ceramics, and coatings/fabrics. It emphasizes the necessity of achieving a comprehensive balance among multi-energy shielding performance, suppression of secondary γ-rays, mechanical and formability properties, and sustainability.

Material selection continues to prioritize high atomic number (Z) and density, utilizing multi-element combinations and complementary absorption edges to cover dominant interaction mechanisms across low-to-high energy ranges, thereby reducing the half-value layer and increasing the mass attenuation coefficient. At the same volume fraction, nanofillers often demonstrate superior mass attenuation coefficients and smaller half-value layers compared to their micro-sized counterparts, though they are more prone to agglomeration and interface compatibility issues.

Building on this, the review discusses synergistic design strategies from three perspectives: composition, structure, and morphology. These include multi-element formulations leveraging K, L, and M absorption-edge synergy; multiscale coupling of micro/nanofillers with multilayer/gradient structures and fibrous networks; and interface engineering achieved through core–shell structures, surface functionalization, and dispersion techniques. Literature comparisons indicate that multilayer/gradient structures and fibrous/fabric networks help maintain attenuation efficiency while suppressing secondary γ-rays and preserving flexibility. In polymer, rubber, and fiber-reinforced systems, the incorporation of high-Z nanoparticles, nanosheets, and nanofibers presents a highly promising platform for developing flexible, wearable, and lead/lead-free shielding.

Furthermore, the review summarizes the application of representative Monte Carlo programs and databases—such as WinXCOM, XCOM, MCNPX, Geant4, and Phy-X/PSD—in γ-ray shielding research. In most systems, simulated parameters like the mass attenuation coefficient and half-value layer show good agreement with experimental results, validating the effectiveness of the “experimental-simulation synergy” in shortening development cycles, reducing irradiation experiment risks, and improving data comparability across studies.

Synthesizing existing findings, it is evident that a linear approach of merely increasing high-Z content is insufficient to meet engineering demands. A more promising pathway involves optimizing γ-ray attenuation efficiency, specific mass, flexibility/strength, and process feasibility under given areal density and thickness constraints. This is achieved through absorption-edge synergy, multi-element formulations, multiscale structural design, and effective dispersion and interface control, laying the foundation for next-generation flexible, wearable, and lead-free shielding materials tailored for aerospace and medical applications.

## 6. Perspectives

In recent years, high-Z nano/micro shielding materials have made substantial progress, and the research focus is gradually shifting from solely pursuing high MAC/low HVL to comprehensively balancing attenuation efficiency, areal density, and secondary-radiation suppression in specific application scenarios. First, from an application perspective, deep-space protection, fixed medical shielding and wearable/flexible protection correspond to different “optimal solutions”: deep-space structures, under stringent mass constraints, must prioritize areal density and secondary radiation, and can adopt gradient Z-sequence multilayer structures that combine high-Z/rare-earth layers with hydrogen-rich polymers and B/Li-containing layers; fixed medical shielding pays more attention to lead-free operation and engineering economy in specific energy ranges, and can achieve stable bulk attenuation at acceptable areal densities through K-edge synergistic doping of W/Gd and similar elements, together with heavy-aggregate concretes, shielding glasses and related systems; wearable/flexible systems need to maximize attenuation per unit areal density at very low areal mass while introducing combinations of self-healing or shape-memory polymers with nanofillers to maintain effective HVL under dynamic bending and minor damage. Based on the content of this review and the latest literature, future research can focus on the following aspects:

(1) Holistic design for multi-energy ranges and secondary-radiation suppression

Existing work mostly evaluates material performance in limited energy ranges or under a single γ source, and lacks “multi-energy integrated” design and validation for deep-space, reactor, or high-energy medical environments. In the future, it is necessary to introduce the concepts of energy-range allocation and absorption-edge synergy at the beginning of material design: reasonably combining Pb/Bi/W and rare-earth elements according to the target spectrum, using staggered K/L absorption edges to cover low-, medium- and high-energy regions, and achieving “hard–soft” matching through multilayer/gradient structures to simultaneously suppress primary and secondary γ rays. Complementarily, based on experiments, programs such as Geant4, MCNPX, and FLUKA should be used more extensively for three-dimensional modeling and dose evaluation of complex structures, rather than being limited to comparisons of average attenuation coefficients of bulk materials.

(2) Multiscale structures and multiphysics coupled properties

According to the results summarized in this review, most systems are still confined to “particle-filled bulk/film” structures. In the future, research on multiscale structures such as core–shell particles, aligned fiber networks, porous/honeycomb structures, and layered gradients can be strengthened to achieve synergistic optimization of shielding, mechanical, thermal-conductive, and fatigue-resistance properties. By tailoring the structure, γ-ray attenuation can be significantly enhanced while improving thermal conductivity and thermal stability, providing examples for integrated thermal management and shielding. Such “structure–function integrated” multiphysics coupled designs are expected to play a role in scenarios such as spacesuits, cabin walls, and electronic packaging.

(3) Flexible, wearable, and reconfigurable systems

Polymers, rubbers, and fiber fabrics remain the main carriers for wearable γ shielding, but the rigidity increase and fatigue problems caused by high filler loadings have not yet been systematically solved. In the future, on the one hand, it is necessary to develop functional fillers with lower density that combine high-Z content and flexibility (such as hollow/porous particles, high-aspect-ratio nanosheets and nanofiber networks); on the other hand, the concept of paraffin-based moldable shielding composites can be adopted to achieve “hand-warm moldability and local reconfiguration” while maintaining high attenuation efficiency, thereby providing new configuration space for complex curved surfaces and individualized protective components. Compared with traditional thermoplastic/thermoset polymers, such “low-melting-point, manually reconfigurable” soft shielding systems are expected to show advantages in emergency reinforcement, temporary protection, and in-orbit repair.

(4) Sustainable manufacturing, data-driven approaches, and standardized evaluation systems

At present, most studies still discuss “green fabrication” and “full life cycle” only qualitatively. In the future, it is necessary to establish quantitative evaluation systems for sustainability and recyclability, and closely link them with data-driven design and standardized testing: on the one hand, visible-light microscopy and image-processing methods can be used to quantitatively characterize the distribution and area fraction of high-Z particles in shielding composites before and after recycling, evaluate the influence of the recycling process on microstructure and attenuation performance, and, combined with long-term service data such as mechanical fatigue, aging and irradiation damage, construct a multidimensional evaluation space of “shielding performance–mechanical reliability–recyclability”. On the other hand, unified databases and open data formats can be used to standardize the parameters and processing information reported in different studies, and, in combination with machine-learning and high-throughput simulations, to mine hidden relationships between composition, structure and properties for rapid screening of candidate materials for specific energy spectra and application scenarios; at the same time, standardized testing and evaluation systems for aerospace, medical protection and nuclear facilities should be gradually established, improving the lateral comparability between different materials and studies.

(5) Interdisciplinary collaboration and engineering demonstration

The ultimate goal is to realize γ shielding materials that “behave like heavy metals towards photons, like textiles towards the human body, and are recyclable towards the environment.” This goal cannot be achieved by a single element or a single process, but requires long-term collaboration across multiple disciplines such as materials science, physics, simulation, manufacturing, and environmental science, ranging from irradiation-damage mechanisms and interfacial electronic structures, to 3D printing, textile integration, spraying, and large-area forming, and further to in-service online monitoring and repair. Only when these links are gradually established will the current lightweight, flexible, lead-free shielding samples in the laboratory have the opportunity to move towards large-scale production, holds the potential to achieve system-level improvements well beyond 50% in specific energy ranges under fixed areal-density constraints, and even to approach an order-of-magnitude efficiency gain under certain local operating conditions, and become routine components in aerospace and medical systems.

## Figures and Tables

**Table 1:** K-shell absorption-edge values of selected metals and rare-earth elements.

Element	Density (g·cm^−3^)	K-Shell Absorption Edge (keV)
Fe	7.87	7.13
Ba	3.51	37.44
La	6.70	38.93
Ce	6.77	40.45
Ta	16.65	67.4
W	19.35	69.53
Pb	11.35	88
Bi	9.35	90.35

**Table 2:** Differences in shielding coefficients of different polymers.

Polymer Matrix	γ-ray Energy E (MeV)	Mass Attenuation Coefficient μ/ρ (cm^2^·g^−1^)	Linear Attenuation Coefficient μ (cm^−1^)	HVL (cm)	TVL (cm)
PAN [6,52]	0.662	0.078	0.1003	6.91	22.96
Nylon 6, PA-6 [52]			0.0976	7.10	23.60
PANI [52]			0.1135	6.11	20.29
PEA [6]		0.080			
PET [6,52]		0.078	0.1098	6.31	20.97
PTFE [52]			0.1609	4.31	14.31
POM [6]		0.079			
PPM [6]		0.082			
PPs [52]			0.1061	6.53	21.71
PPy [52]			0.1235	5.61	18.65
NR [6]		0.084			
LDPE [16]	0.015		0.0169		

**Table 4:** Shielding coefficients of composite materials with different fillers added to resin and rubber matrices.

Polymer Matrix	Filler Type	Filler Loading (wt.%)	γ-Ray Energy E (MeV)	Mass Attenuation Coefficient μ/ρ (cm^2^·g^−1^)	Linear Attenuation Coefficient μ (cm^−1^)	HVL (cm)
Epoxy resin [80]	Y_2_O_3_ + GeO_2_ Nps	9.55 + 8.42	0.6638	1.050	1.439	0.48
Epoxy wall point [82]	Bi_2_O_3_ micro	20	0.662	0.0864		
NR/SBR [85]	PbS	500 phr			29.5	0.023
NR [86]	BaWO_4_ micro	100 phr		0.09	0.307	2.258
Silicone–rubber [90]	Bi_2_O_3_ Nps	20	0.060		3.55	0.2
Silicone–rubber [91]	WO_3_ micro	70	0.662	0.072	0.252	2.74
RTV silicone–rubber [92]	W + Bi_2_O_3_ micro	18.75 + 18.75	0.779		0.0059	0.117

**Table 5:** Shielding coefficients of polyolefin composites with different fillers.

Polymer Matrix	Filler Type	Filler Loading (wt.%)	γ-Ray Energy E (MeV)	Mass Attenuation Coefficient μ/ρ (cm^2^·g^−1^)	Linear Attenuation Coefficient μ (cm^−1^)	HVL (cm)
HDPE [93]	PbO-NPs	50	0.662	0.114	0.189	3.67
HDPE [99]	ZnO-NPs	40		0.099	0.121	5.728
HDPE [100]	B_4_C + Fe	10 + 30		0.0823	0.113	0.12
HDPE [103]	Gd_2_O_3_-NPs + B_4_C	10 + 20		0.07	0.088	7.9
r-HDPE [94]	PbO-NPs	50		0.114	0.189	3.67
R-HDPE [107]	CuO-NPs + PTA	40		0.099	0.124	5.594
LLDPE [102]	W–BNNS	20		0.093	0.093	7.45
LLDPE [106]	PbO micro	30		0.099	0.124	5.594
UHMWPE [96]	Bi_2_O_3_-NPs	2	0.3839	0.117	0.124	5.59
UHMWPE [97]	W-NPs	60	0.122		3.43	0.2
PP [109]	Bi_2_O_3_-NPs	50	0.06		5.59	0.124
PP [111]	CdO-NPs	40		2.29188	3.29	0.211

**Table 6:** Radiation-shielding coefficient values of Different glass systems.

Glass System	Main High-Z Element(s)	γ-Ray Energy E (MeV)	Mass Attenuation Coefficient μ/ρ (cm^2^·g^−1^)	Linear Attenuation Coefficient μ (cm^−1^)	HVL (cm)
Li_2_O–PbO–Bi_2_O_3_–B_2_O_3_ [169]	Pb, Bi	0.5	0.153	0.91	0.76
BaO–TeO_2_–B_2_O_3_ glass-ceramic [170]	Bi, Ba, Te	0.511		0.64	1.082
Bi_2_O_3_–TeO_2_–Li_2_O–Al_2_O_3_ [171]	Bi, Te	0.662	0.08	0.38	1.58
BaO-containing borosilicate glass-ceramic [172]	Ba	0.059		9.497	0.073
Bi_2_O_3_/BaO-modified borosilicate glass [176]	Bi, Ba	0.662		0.246–0.365	1.90
ZnO–B_2_O_3_–TeO_2_–WO_3_ [179]	W, Te, Zn	0.511		0.486	2.07
BaTiO_3_–B_2_O_3_–MgO–Na_2_O–CaO [180]	Ba, Ti	0.276		0.3475	
Na_2_Si_3_O_7_/Ag glassy composites [181]	Ag	0.14	0.296		
Silicate glass with Bi_2_O_3_ micro- and nanoparticles [182]	Bi	0.5953	1.19		

## Data Availability

No new data were created or analyzed in this study.

## Nomenclature

ALARA	as low as reasonably achievable
IRPA	International Radiation-Protection Association
MCNPX	Monte Carlo N-Particle eXtended
HVL	half-value layer
SEM	scanning electron microscopy
EGSnrc	electron–gamma shower code (nrc)
EP	epoxy resin
Phy-X/PSD	Photon-Shielding Database
WinXCOM	XCOM photon cross-section database (Windows)
SEBS	styrene–ethylene–butylene–styrene
HPGe	high-purity germanium detector
LDPE	low-density polyethylene
HDPE	high-density polyethylene
LLDPE	linear low-density polyethylene
PVA	poly(vinyl alcohol)
UHDPE	ultra-high-density polyethylene
UPR	unsaturated polyester resin
ISO	isophthalic unsaturated polyester
MAC	mass attenuation coefficient
RPE	radiation-protection efficiency
CNT	carbon nanotube
NBR	nitrile-butadiene rubber
CR	chloroprene rubber
EDS	energy-dispersive X-ray spectroscopy
UHMWPE	ultra-high-molecular-weight polyethylene
BNNS	boron nitride nanosheets
EPM	ethylene–propylene rubber
TEM	transmission electron microscopy
PTA	phosphotungstic acid
MCNP5	Monte Carlo N-Particle version 5
BVO	bismuth vanadate
LAC	linear attenuation coefficient
PMMA	poly(methyl methacrylate)
EABF	energy absorption buildup factor
EBF	exposure buildup factor
CMT	calcium borate
PI	polyimide
HVCMC	high-volume-fraction composite
PPVE	poly(vinyl ester)
ABS	acrylonitrile–butadiene–styrene
GMASS	tungsten-based thermoplastic composite
PEEK	poly(ether ether ketone)
FDM	fused deposition modeling
OPC	ordinary Portland cement
NF	nanofiller
WOPC	white ordinary Portland cement
TVL	tenth-value layer
MFP	mean free path
NC	normal concrete
BC	barite concrete
SC	siderite concrete

## References

[1] Pan Z.Q. (1996). Basic structures for strengthening radiation protection. Radiat. Prot..

[2] Schaeffer N.M. (1973). Reactor Shielding for Nuclear Engineers.

[3] Chen J.Y., Zhao Y.C. (2014). Research advances on risk models of radiogenic cancer and risk transfer models between populations. Int. J. Radiat. Med. Nucl. Med..

[4] Sun Z.J., Wang J.X., Xiang J., Zhao Y.C., Chen J.Y., Yang Q.Q., Fan S.J. (2015). Estimation of radiation-induced gastric cancer risk coefficients in the Chinese population. Chin. J. Radiol. Med. Prot..

[5] Akman F., Sayyed M.I., Kaçal M.R., Tekin H.O. (2019). Investigation of photon shielding performances of some selected alloys by exper imental data, theoretical and MCNPX code in the energy range of 81 keV–1333 keV. J. Alloys Compd..

[6] Bhosale R.R., More C.V., Gaikwad D.K., Pawar P.P., Rode M.N. (2017). Radiation shielding and gamma ray attenuation properties of some polymers. Nucl. Technol. Radiat. Prot..

[7] Slaba T.C., Bahadori A.A., Reddell B.D., Singleterry R.C., Clowdsley M.S., Blattnig S.R. (2017). Optimal shielding thickness forgalactic cosmic ray environments. Life Sci. Space Res..

[8] More C.V., Alsayed Z., Badawi M.S., Thabet A.A., Pawar P.P. (2021). Polymeric composite materials for radiation shielding: A review. Environ. Chem. Lett..

[9] Wu S., Zhang W., Yang Y. (2024). Progress in flexible and wearable lead-free polymer composites for radiation protection. Polymers.

[10] Shahzad K., Kausar A., Manzoor S., Rakha S.A., Uzair A., Sajid M., Arif A., Khan A.F., Diallo A., Ahmad I. (2022). Views on radiation shielding efficiency of polymeric nanocomposites for aerospace struc tural applications. Radiation.

[11] Wang B., Qiu T., Yuan L., Fang Q., Wang X., Guo X., Zhang D., Lai C., Wang Q., Liu Y. (2023). A comparative study between pure bismuth/tungsten and the bismuth tungsten oxide for flexible shielding of gamma/X rays. Radiat. Phys. Chem..

[12] Asgari M., Afarideh H., Ghafoorifard H., Amirabadi E.A. (2021). Comparison of nano/micro lead, bismuth and tungsten on the gamma shielding properties of the flexible composites against photon in wide energy range (40 keV–662 keV). Nucl. Eng. Technol..

[13] Wu H.X. (2005). Study on Nano-Lead Composite Materials for High-Energy Radiation Protection. Master’s Thesis.

[14] Alsaab A.H., Zeghib S. (2023). Study of prepared lead-free polymer nanocomposites for X- and gamma-ray shielding in healthcare applications. Polymers.

[15] Hubbell J.H. (1999). Review of photon interaction cross section data in the medical and biological context. Phys. Med. Biol..

[16] Wang B., Guo X., Yuan L., Fang Q., Wang X., Qiu T., Lai C., Wang Q., Liu Y. (2023). Micro gadolinium oxide dispersed flexible composites developed for the shielding of thermal neutron/gamma rays. Nucl. Eng. Technol..

[17] Almisned G., Tekin H.O., Kavaz E., Bilal G., Issa S.A., Zakaly H.M., Ene A. (2021). Gamma, Fast Neutron, Proton, and Alpha Shielding Properties of Borate Glasses: A Closer Look on Lead (II) Oxide and Bismuth (III) Oxide Reinforcement. Appl. Sci..

[18] Jia X.B. (2016). The Preparation of La/Ce-Doped Ba_5_Ta_4_O_15_ and its Gamma-Ray Shielding Properties of NR Composites. Master’s Thesis.

[19] Shen X.H. (2008). Preparation of Polymer/Lead Radiation-Protection Materials and Their Radiation Shielding Properties. Master’s Thesis.

[20] Li H.F. (2009). Preparation and Performance Study of Lead-Containing Radiation-Protective Acrylic (PMMA) Plates. Master’s Thesis.

[21] Kua A., Ogunseitan O., Saphores J.D., Shapiro A.A., Schoenung J.M. Lead-Free Solders: Issues of Toxicity, Availability and Impacts of Extraction. Proceedings of the 53rd Electronic Components and Technology Conference (ECTC) 2003.

[22] He D.Y. (2022). Life-Cycle Designed Hybrid Nanofibrous Membranes for X-Ray Protection and Photocatalysis. Ph.D. Thesis.

[23] Chandrika B., Ambika M., Seenappa L., Manjunatha H., Sridhar K., Lourduraj A.C., Kuttukaran S.S. (2024). Gamma shielding ability of Bismuth–Tungsten oxide (Bi_2_O_6_W) nanoparticles. Radiat. Phys. Chem..

[24] Li Y., Wang G.W., Wu Z.X., Zhang H.T., Liu G.Q. (2023). Study on Gamma Ray Shielding Properties of Bismuth Tungstate with Different Morphologies. J. Isot..

[25] Li R., Gu Y., Wang Y., Yang Z., Li M., Zhang Z. (2017). Effect of particle size on gamma radiation shielding property of gadolinium oxide dispersed epoxy resin matrix composite. Mater. Res. Express.

[26] Yao Y. (2017). Design and Evaluation of Γ-Ray Shielding Materials Doped with Bismuth Oxide. Master’s Thesis.

[27] Wang J., Wang S., Xu Z.L., Gao Y., You Z.M., Fan J.K., Du X., Zhang Q. (2020). Silicone-Based Flexible Shielding Material for Gamma-Ray Shielding and Its Preparation Method. Chinese Patent.

[28] Wang J., Wang S., Zhou H., Wu Y., Gao Y., Hu Y., Xu Z., Zhang J., Fan J., You Z. (2020). Flexible Silicone-Based Material Doped with Nano Titanium Oxide for Gamma-Ray Shielding and Its Preparation Method. Chinese Patent.

[29] Xu F., Xu J., Qiu Q., Zhou Y. (2016). Gamma-Ray Shielding Fabric. Chinese Patent.

[30] Wang B., Wu S., Liu Y., Huang Q., Qin E., Wei S., Pan C., Lu H., Shi Y., Ma X. (2023). Preparation Method of Gamma-Ray Shielding Coating. Chinese Patent.

[31] Azman M.N., Abualroos N.J., Yaacob K.A., Zainon R. (2023). Feasibility of nanomaterial tungsten carbide as lead-free nanomaterial-based radiation shielding. Radiat. Phys. Chem..

[32] Gulbicim H., Aksu M., Turkan N., Babuz C. (2023). Gamma ray shielding properties of synthesized lanthanide borides using EGSnrc code. Nucl. Instrum. Methods Phys. Res. Sect. B Beam Interact. Mater. At..

[33] Wei W., Yang H., Yuan Y., Li Y., Cui K., Zhang T., Jia X., Qin W., Wu X. (2023). Enhanced wide energy regions gamma ray shielding property for Bi_2_O_3_–Gd_2_O(CO_3_)_2_·H_2_O/EP composites with strong electron cloud overlap. J. Alloys Compd..

[34] Zhao Y. (2017). Controllable Synthesis of Bismuth Tungstate Nanocrystal and Its γ-Ray Shielding Properties. Master’s Thesis.

[35] Wang G.W., Zhang H.T., Mao C.Y., Wu Z.X., Liu Y., Yan Q., Liu G.Q. (2023). Study on the shielding property of Er^3+^-doped Bi_2_WO_6_ against low-energy gamma rays. Nucl. Electron. Detect. Technol..

[36] Li J.S., Dai Y.D., Zhang Y., Sun H., Chang S.Q. (2011). Preparation process and properties of samarium polyacrylate/epoxy resin radiation-protection materials. At. Energy Sci. Technol..

[37] Wani A.L., Ara A., Usmani J.A. (2015). Lead toxicity: A review. Interdiscip. Toxicol..

[38] Bača P., Vanýsek P. (2023). Issues concerning manufacture and recycling of lead. Energies.

[39] May G.J., Davidson A., Monahov B. (2018). Lead batteries for utility energy storage: A review. J. Energy Storage.

[40] Yanamandra K., Pinisetty D., Daoud A., Gupta N. (2022). Recycling of Li-ion and lead acid batteries: A review. J. Indian Inst. Sci..

[41] Mohan R. (2010). Green bismuth. Nat. Chem..

[42] Nassar N.T., Graedel T.E., Harper E.M. (2015). By-product metals are technologically essential but have problematic supply. Sci. Adv..

[43] Graedel T.E., Harper E.M., Nassar N.T., Nuss P., Reck B.K., Turner B.L. (2015). Criticality of metals and metalloids. Proc. Natl. Acad. Sci. USA.

[44] Tang L., Wang P., Graedel T.E., Pauliuk S., Xiang K., Ren Y., Chen W. (2020). Refining the understanding of China’s tungsten dominance with dynamic material cycle analysis. Resour. Conserv. Recycl..

[45] Jowitt S.M. (2018). Introduction to a Resources Special Issue on criticality of the rare earth elements: Current and future sources and recycling. Resources.

[46] Alshahri S., Alsuhybani M., Alosime E., Almurayshid M., Alrwais A., Alotaibi S. (2021). LDPE/Bismuth Oxide Nanocomposite: Preparation, Characterization and Application in X-ray Shielding. Polymers.

[47] Marshall S.K., Boonpeng K., Buapud N., Chimhashat S., Chuaymuang J., Kwandee P., Songphum N. (2025). Bismuth Oxide Nanoparticle-Enhanced Poly(methyl methacrylate) Composites for I-131 Radiation Shielding: A Combined Simulation and Experimental Investigation. Polymers.

[48] Hubbell J.H. (2006). Review and history of photon cross section calculations. Phys. Med. Biol..

[49] Şakar E., Özpolat Ö.F., Alım B., Sayyed M.I., Kurudirek M. (2020). Phy-X/PSD: Development of a user friendly online software for calculation of parameters relevant to radiation shielding and dosimetry. Radiat. Phys. Chem..

[50] Agostinelli S., Allison J., Amako K., Apostolakis J., Araujo H., Arce P., Asai M., Axen D., Banerjee S., Barrand G. (2003). GEANT4—A simulation toolkit. Nucl. Instrum. Methods Phys. Res. Sect. A.

[51] Briesmeister J.F. (2000). MCNP—A General Monte Carlo N-Particle Transport Code, Version 4C.

[52] Kaçal M.R., Akman F., Sayyed M.I. (2019). Evaluation of gamma-ray and neutron attenuation properties of some polymers. Nucl. Eng. Technol..

[53] Kilicoglu O., Kara U., Inanc I. (2021). The impact of polymer additive for N95 masks on gamma-ray attenuation properties. Mater. Chem. Phys..

[54] Mann H.S., Brar G.S., Mudahar G.S. (2016). Gamma-ray shielding effectiveness of novel light-weight clay-flyash bricks. Radiat. Phys. Chem..

[55] Sharma A., Sayyed M.I., Agar O., Kaçal M.R., Polat H., Akman F. (2020). Photon-shielding performance of bismuth oxychloride-filled polyester concretes. Mater. Chem. Phys..

[56] Biswas R., Sahadath H., Mollah A.S., Huq M.F. (2016). Calculation of gamma-ray attenuation parameters for locally developed shielding material: Polyboron. J. Radiat. Res. Appl. Sci..

[57] Harish V., Nagaiah N., Prabhu T.N., Varughese K.T. (2009). Preparation and characterization of lead monoxide filled unsaturated polyester based polymer composites for gamma radiation shielding applications. J. Appl. Polym. Sci..

[58] Yazdani-Darki S., Eslami-Kalantari M., Feizi S., Zare H. (2023). Study of the structural and shielding properties of unsaturated polyester/lead oxide nanocomposites against gamma-rays. Radiat. Phys. Chem..

[59] Harish V., Nagaiah N., Kumar H.G. (2012). Lead oxides filled isophthalic resin polymer composites for gamma radiation shielding applications. Indian J. Pure Appl. Phys..

[60] Mirji R., Lobo B. (2020). Study of polycarbonate–bismuth nitrate composite for shielding against gamma radiation. J. Radioanal. Nucl. Chem..

[61] Ghule P.G., Bholane G.T., Joshi R.P., Dahiwale S.S., Shelke P.N., Dhole S.D. (2024). Gamma radiation shielding properties of unsaturated polyester/Bi_2_O_3_ composites: An experimental, theoretical and simulation approach. Radiat. Phys. Chem..

[62] Yılmaz M., Akman F. (2023). Gamma radiation shielding properties for polymer composites reinforced with bismuth tungstate in different proportions. Appl. Radiat. Isot..

[63] Ambika M.R., Nagaiah N., Suman S.K. (2017). Role of bismuth oxide as a reinforcer on gamma shielding ability of unsaturated polyester based polymer composites. J. Appl. Polym. Sci..

[64] Akman F., Ogul H., Kaçal M.R., Polat H., Dilsiz K., Agar O. (2021). Gamma attenuation characteristics of CdTe-Doped polyester composites. Prog. Nucl. Energy.

[65] Bagheri K., Razavi S.M., Ahmadi S.J., Kosari M., Abolghasemi H. (2018). Thermal resistance, tensile properties, and gamma radiation shielding performance of unsaturated polyester/nanoclay/PbO composites. Radiat. Phys. Chem..

[66] Hemily H.M., Saleh I.H., Ghataas Z.F., Abdel-Halim A.A., Hisam R., Shah A.Z., Sayyed M.I., Yasmin S., Elsafi M. (2022). Radiation shielding enhancement of polyester adding artificial marble materials and WO_3_ nanoparticles. Sustainability.

[67] Elsafi M., Sayyed M.I., Almuqrin A.H. (2024). New polyester composites synthesized with additions of different sized ZnO to study their shielding efficiency. Nucl. Eng. Technol..

[68] Oğul H., Agar O., Bulut F., Kaçal M.R., Dilsiz K., Polat H., Akman F. (2023). A comparative neutron and gamma-ray radiation shielding investigation of molybdenum and boron filled polymer composites. Appl. Radiat. Isot..

[69] Oğul H., Polat H., Akman F., Kaçal M.R., Dilsiz K., Bulut F., Agar O. (2022). Gamma and neutron shielding parameters of polyester-based composites reinforced with boron and tin nanopowders. Radiat. Phys. Chem..

[70] Başgöz Ö., Güler S.H., Güler Ö., Canbay C.A., Zakaly H.M., Issa S.A., Almisned G., Tekin H. (2022). Synergistic effect of boron nitride and graphene nanosheets on behavioural attitudes of polyester matrix: Synthesis, experimental and Monte Carlo simulation studies. Diam. Relat. Mater..

[71] Elmahroug Y., Tellili B., Souga C. (2014). Determination of shielding parameters for different types of resins. Ann. Nucl. Energy.

[72] Dong M.G., Xue X.X., Yang H., Li Z.F. (2016). Shielding analysis and irradiation resistant effect of epoxy resin for gamma ray. At. Energy Sci. Technol..

[73] Chang L., Zhang Y., Liu Y., Fang J., Luan W., Yang X., Zhang W. (2015). Preparation and characterization of tungsten/epoxy composites for γ-rays radiation shielding. Nucl. Instrum. Methods Phys. Res. Sect. B Beam Interact. Mater. At..

[74] Li J.S., Dai Y.D., Zhang Y., Sun H., Chang S.Q. (2010). Preparation of Er_2_O_3_/epoxy resin composite material for radiation-protection and its property research. New Chem. Mater..

[75] Erdogan F., Goddard B., Mohammadi R., Rojas J.V. (2024). Gamma-ray and neutron attenuation of hafnium diboride-epoxy composites. Radiat. Phys. Chem..

[76] Al-Sarray E., Akkurt I., Gunoglu K., Evcin A., Bezir N.C. (2017). Radiation shielding properties of some composite panel. Acta Phys. Pol. A.

[77] Aldhuhaibat M.J.R., Amana M.S., Jubier N.J., Salim A.A. (2021). Improved gamma radiation shielding traits of epoxy composites: Evaluation of mass attenuation coefficient, effective atomic and electron number. Radiat. Phys. Chem..

[78] Sayyed M.I., Yasmin S., Almousa N., Elsafi M. (2022). Shielding Properties of Epoxy Matrix Composites Reinforced with MgO Micro- and Nanoparticles. Materials.

[79] Zhang H.X. (2014). Preparation and Study on Functional Carbon Nanotubes/Epoxy Radiation Shielding Composite Materials. Master’s Thesis.

[80] Dai X.Z., Xiao D.T. (2016). Preparation of Y_2_O_3_/GeO_2_/epoxy resin-based multilayer radiation shielding material and its property research. J. Univ. South China (Sci. Technol.).

[81] Alavian H., Samie A., Tavakoli-Anbaran H. (2020). Experimental and Monte Carlo investigations of gamma ray transmission and buildup factors for inorganic nanoparticle/epoxy composites. Radiat. Phys. Chem..

[82] Vishnu C.V., Joseph A., Anju K. (2023). Evaluation of the gamma radiation shielding characteristics of epoxy wall paint modified with micro sized Bi_2_O_3_ and WO_3_. Mater. Today Proc..

[83] Abualroos N.J., Yaacob K.A., Zainon R. (2023). Radiation attenuation effectiveness of polymer-based radiation shielding materials for gamma radiation. Radiat. Phys. Chem..

[84] Dong Y., Dai Y.D., Chang S.Q., Kang B., Yang X.Y. (2012). Preparation of WO_3_/CeO_2_/epoxy resin-based multilayer radiation shielding material and its property research. Mater. Rev..

[85] Gwaily S.E. (2002). Galena/(NR+SBR) rubber composites as gamma radiation shields. Polym. Test..

[86] Vishnu C.V., Joseph A. (2024). Gamma-ray shielding analysis on natural rubber composites fortified with barium tungstate (BaWO_4_). Radiat. Phys. Chem..

[87] Aziz O., Salama E., El-Nashar D.E., Bakry A. (2023). Development of sustainable radiation-shielding blend using natural rubber/NBR, and bismuth filler. Sustainability.

[88] Liang F. (2015). Preparation of Bi_3_B_5_O_12_ and Its Synergistic Neutron–Gamma-Ray Shielding Performance. Ph.D. Thesis.

[89] Malekie S., Hajiloo N. (2017). Comparative study of micro and nano size WO_3_/E_44_ epoxy composite as gamma radiation shielding using MCNP and experiment. Chin. Phys. Lett..

[90] Almuqrin A.H., Sayyed M.I., Elsafi M., Khandaker M.U. (2022). Comparison of radiation shielding ability of Bi_2_O_3_ micro and nanoparticles for radiation shields. Radiat. Phys. Chem..

[91] Zali V.S., Jahanbakhsh O., Ahadzadeh I. (2022). Preparation and evaluation of gamma shielding properties of silicon-based composites doped with WO_3_ micro- and nanoparticles. Radiat. Phys. Chem..

[92] Atashi P., Rahmani S., Ahadi B., Rahmati A. (2018). Efficient, flexible and lead-free composite based on room temperature vulcanizing silicone rubber/W/Bi_2_O_3_ for gamma ray shielding application. J. Mater. Sci. Mater. Electron..

[93] Mahmoud M.E., El-Khatib A.M., Badawi M.S., Rashad A.R., El-Sharkawy R.M., Thabet A.A. (2018). Fabrication, characterization and gamma rays shielding properties of nano and micro lead oxide-dispersed-high density polyethylene composites. Radiat. Phys. Chem..

[94] Mahmoud M.E., El-Khatib A.M., Badawi M.S., Rashad A.R., El-Sharkawy R.M., Thabet A.A. (2018). Recycled high-density polyethylene plastics added with lead oxide nanoparticles as sustainable radiation shielding materials. J. Clean. Prod..

[95] Harrison C., Burgett E., Hertel N., Grulke E. (2008). Polyethylene/boron composites for radiation shielding applications. AIP Conference Proceedings (Space Technology and Applications International Forum—STAIF-2008).

[96] Abdalsalam A.H., Sayyed M.I., Hussein T.A., Şakar E., Mhareb M.H.A., Şakar B.C., Alim B., Kaky K.M. (2019). A study of gamma attenuation property of UHMWPE/Bi_2_O_3_ nanocomposites. Chem. Phys..

[97] Kaloshkin S.D., Tcherdyntsev V.V., Gorshenkov M.V., Gulbin V.N., Kuznetsov S.A. (2012). Radiation-protective polymer-matrix nanostructured composites. J. Alloys Compd..

[98] Alsayed Z., Badawi M.S., Awad R., El-Khatib A.M., Thabet A.A. (2020). Investigation of γ-ray attenuation coefficients, effective atomic number and electron density for ZnO/HDPE composite. Phys. Scr..

[99] Alsayed Z., Badawi M.S., Awad R., Thabet A.A., El-Khatib A.M. (2019). Study of some γ-ray attenuation parameters for new shielding materials composed of nano ZnO blended with high density polyethylene. Nucl. Technol. Radiat. Prot..

[100] Reda A.M., Alsawah M.A., Hosni M., Ahmed R.M. (2024). Gamma-ray shielding effectiveness, thermal, and dielectric properties of filler-reinforced high-density polyethylene/boron carbide composites. Prog. Nucl. Energy.

[101] Hosseini M.A., Malekie S., Keshavarzi M. (2021). Analysis of radiation shielding characteristics of magnetite/high density polyethylene nanocomposite at diagnostic level using the MCNPX, XCOM, XMuDat and Auto-Zeff programs. Mosc. Univ. Phys. Bull..

[102] Kim S., Ahn Y., Song S.H., Lee D. (2022). Tungsten nanoparticle anchoring on boron nitride nanosheet-based polymer nanocomposites for complex radiation shielding. Compos. Sci. Technol..

[103] Zhao S., Huo Z.P., Zhong G.Q., Zhang H., Hu L.Q. (2022). Preparation of modified gadolinium/boron/polyethylene nanocomposites and their shielding properties for neutron and gamma rays. Chem. J. Chin. Univ..

[104] Kim J., Seo D., Lee B.C., Seo Y.S., Miller W.H. (2014). Nano-W dispersed gamma radiation shielding materials. Adv. Eng. Mater..

[105] Olukotun S.F., Gbenu S.T., Oladejo O.F., Sayyed M.I., Tajudin S.M., Amosun A., Fadodun O., Fasasi M. (2020). Investigation of gamma ray shielding capability of fabricated clay-polyethylene composites using EGS5, XCOM and Phy-X/PSD. Radiation Physics and Chemistry.

[106] Belgin E.E., Aycik G.A. (2015). Preparation and radiation attenuation performances of metal oxide filled polyethylene based composites for ionizing electromagnetic radiation shielding applications. J. Radioanal. Nucl. Chem..

[107] El-Taher A., Zakaly H.M.H., Pyshkina M., Allam E.A., El-Sharkawy R.M., Mahmoud M.E., Abdel-Rahman M.A.E. (2021). A comparative Study Between Fluka and Microshield Modeling Calculations to study the Radiation-Shielding of Nanoparticles and Plastic Waste composites. Z. Für Anorg. Und Allg. Chem..

[108] El-Khatib A.M., Abbas M.I., Elzaher M.A., Badawi M.S., Alabsy M.T., Alharshan G.A., Aloraini D.A. (2019). Gamma attenuation coefficients of nano cadmium oxide/high density polyethylene composites. Sci. Rep..

[109] El-Khatib A.M., Shalaby T.I., Antar A., Elsafi M. (2022). Experimental study of polypropylene with additives of Bi_2_O_3_ nanoparticles as radiation-shielding materials. Polymers.

[110] El-Khatib A.M., Shalaby T.I., Antar A., Elsafi M. (2022). Improving Gamma Ray Shielding Behaviors of Polypropylene Using PbO Nanoparticles: An Experimental Study. Materials.

[111] Zakaly H.M.H., Ashry A., El-Taher A., Abbady A.G.E., Allam E.A., El-Sharkawy R.M., Mahmoud M.E. (2021). Role of novel ternary nanocomposites polypropylene in nuclear radiation attenuation properties: In-depth simulation study. Radiat. Phys. Chem..

[112] Kassem S.M., Abdel Maksoud M.I.A., El Sayed A.M., Ebraheem S., Helal A.I., Ebaid Y.Y. (2023). Optical and radiation shielding properties of PVC/BiVO_4_ nanocomposite. Sci. Rep..

[113] Nasrabadi M., Tavakoli-Anbaran H., Ebrahimibasabi E. (2024). Investigation of nano MgO loaded polyvinyl chloride polymer in protective clothing as a nonlead materials. Heliyon.

[114] El-Sharkawy R.M., Abdou F.S., Gizawy M.A., Allam E.A., Mahmoud M.E. (2023). Bismuth oxide nanoparticles (Bi_2_O_3_ NPs) embedded into recycled poly(vinyl chloride) plastic sheets as a promising shielding material for gamma radiation. Radiat. Phys. Chem..

[115] Muthamma M.V., Bubbly S.G., Gudennavar S.B., Narendranath K.C.S. (2019). Poly(vinyl alcohol)–bismuth oxide composites for X-ray and γ-ray shielding applications. J. Appl. Polym. Sci..

[116] Issa S.A.M., Mostafa A.M.A., Hanafy T.A., Dong M., Xue X. (2019). Comparison study of photon attenuation characteristics of poly vinyl alcohol (PVA) doped with Pb(NO_3_)_2_ by MCNP5 code, XCOM and experimental results. Prog. Nucl. Energy.

[117] Kazemi F., Malekie S., Hosseini M.A. (2019). A Monte Carlo study on the shielding properties of a novel polyvinyl alcohol (PVA)/WO_3_ composite, against gamma rays, using the MCNPX code. J. Biomed. Phys. Eng..

[118] Hosseini M.A., Malekie S., Kazemi F. (2022). Experimental evaluation of gamma radiation shielding characteristics of polyvinyl alcohol/tungsten oxide composite: A comparison study of micro and nano sizes of the fillers. Nucl. Instrum. Methods Phys. Res. Sect. A Accel. Spectrometers Detect. Assoc. Equip..

[119] Srinivasan K., Samuel E.J.J. (2017). Evaluation of radiation shielding properties of the polyvinyl alcohol/iron oxide polymer composite. J. Med. Phys..

[120] Issa S.A.M., Zakaly H.M.H., Pyshkina M., Mostafa M.Y.A., Rashad M., Soliman T.S. (2021). Structure, optical, and radiation shielding properties of PVA–BaTiO_3_ nanocomposite films: An experimental investigation. Radiat. Phys. Chem..

[121] Manjunatha H.C. (2017). A study of gamma attenuation parameters in poly methyl methacrylate and Kapton. Radiat. Phys. Chem..

[122] Soni G., Gouttam N., Joshi V. (2022). Synthesis and comparisons of optical and gamma radiation shielding properties for ZnO and SiO_2_ nanoparticles in PMMA nanocomposites thin films. Optik.

[123] Bel T., Arslan C., Baydogan N. (2019). Radiation shielding properties of poly(methyl methacrylate)/colemanite composite for the use in mixed irradiation fields of neutrons and gamma rays. Mater. Chem. Phys..

[124] El-Khatib A.M., Alabsy M.T., El-Khatib A.Y., Dib M.F., Abbas M.I. (2024). Superiority of micro/nano tungsten carbide reinforced poly-methyl methacrylate composites in shielding gamma radiation. Nucl. Eng. Technol..

[125] Saberi Rise M., Hosseini Ranjbar A., Noori H., Saheb V. (2023). Bi-PMMA composite materials and their shielding capability for low energy gamma rays. Radiat. Phys. Chem..

[126] Ismail A.M., Elmoulaa N.B.G., El Shazly R.M., Ashry A. (2023). Impact of BaTiO_3_ on the structural, optical, and nuclear radiation shielding parameters of poly(methyl methacrylate) nanocomposites as transparent shielding material. Radiat. Phys. Chem..

[127] Pavlenko V.I., Cherkashina N.I., Yastrebinsky R.N. (2019). Synthesis and radiation shielding properties of polyimide/Bi_2_O_3_ composites. Heliyon.

[128] Baykara O., İrim Ş.G., Wis A.A., Keskin M.A., Ozkoc G., Avci A., Doğru M. (2020). Polyimide nanocomposites in ternary structure: “A novel simultaneous neutron and gamma-ray shielding material”. Polym. Adv. Technol..

[129] Abdali K. (2022). Structural, morphological, and gamma ray shielding (GRS) characterization of HVCMC/PVP/PEG polymer blend encapsulated with silicon dioxide nanoparticles. Silicon.

[130] Cinan Z.M., Erol B., Baskan T., Mutlu S., Ortaç B., Yilmaz S.S., Yilmaz A.H. (2022). Radiation shielding tests of crosslinked polystyrene-b-polyethyleneglycol block copolymers blended with nanostructured selenium dioxide and boron nitride particles. Nanomaterials.

[131] Turhan M.F., Akman F., Polat H., Kaçal M.R., Demirkol İ. (2020). Gamma-ray attenuation behaviors of hematite doped polymer composites. Prog. Nucl. Energy.

[132] El-Toony M.M., Eid G., Algarni H.M., Alhuwaymel T.F., Abel-hady E.E. (2020). Synthesis and characterisation of smart poly vinyl ester/Pb_2_O_3_ nanocomposite for gamma radiation shielding. Radiat. Phys. Chem..

[133] Ceh J., Youd T., Mastrovich Z., Peterson C., Khan S., Sasser T.A., Sander I.M., Doney J., Turner C., Leevy W.M. (2017). Bismuth infusion of ABS enables additive manufacturing of complex radiological phantoms and shielding equipment. Sensors.

[134] Wu Y., Cao Y., Wu Y., Li D. (2020). Mechanical properties and gamma-ray shielding performance of 3D-printed poly-ether-ether-ketone/tungsten composites. Materials.

[135] Sobczak J., Truszkiewicz A., Korczeniewski E.D., Cyganiuk A., Terzyk A.P., Kolanowska A., Jędrysiak R.G., Boncel S., Żyła G. (2023). Shape-controlled iron–paraffin composites as γ- and X-ray shielding materials formable by warmth-of-hands-derived plasticity. ACS Appl. Eng. Mater..

[136] Sobczak J., Truszkiewicz A., Cwynar K., Ruczka S., Kolanowska A., Jedrysiak R.G., Waśkiewicz S., Dzida M., Boncel S., Żyła G. (2024). Soft, ternary, X- and gamma-ray shielding materials: Paraffin-based iron-encapsulated carbon nanotube nanocomposites. Mater. Adv..

[137] Sobczak J., Truszkiewicz A., Ruczka S., Gancarz P., Cyganiuk A., Fal J., Korczeniewski E., Poręba M., Cwynar K., Marcos M.A. (2025). High-efficient, manually-shapeable gamma- and X-ray shield—An introduction of paraffin-tungsten microcomposite along with its properties and recycling possibilities. Mater. Des..

[138] Sobczak J., Cioch K., Żyła G. (2025). Paraffin-based composites containing high density particles: Lead and bismuth and its’ oxides as γ-ray shielding materials: An experimental study. Discov. Nano.

[139] Hager M.D., Greil P., Leyens C., van der Zwaag S., Schubert U.S. (2010). Self-healing materials: Past, present and future. Adv. Mater..

[140] Leng J., Lan X., Liu Y., Du S. (2011). Shape-memory polymers and their composites: Stimulus methods and applications. Prog. Mater. Sci..

[141] Akkas A., Tugrul A.B., Buyuk B., Addemir A.O., Marsoglu M., Agacan B. (2015). Shielding effect of boron carbide aluminium metal matrix composite against gamma and neutron radiation. Acta Phys. Pol. A.

[142] Singh V.P., Badiger N.M. (2014). Gamma ray and neutron shielding properties of some alloy materials. Ann. Nucl. Energy.

[143] Agar O., Sayyed M.I., Akman F., Tekin H.O., Kaçal M.R. (2019). An extensive investigation on gamma ray shielding features of Pd/Ag-based alloys. Nucl. Eng. Technol..

[144] Majeed E.I., Najam L.A., Hamood M.A., Mahmoud K.A. (2025). A comprehensive study on the γ-ray shielding performance of Al–Cu–PbO alloy: Experimental and simulation studies. Nucl. Eng. Technol..

[145] Ma X.J. (2014). Experimental Study of γ-Ray and Neutron Shielding by Thermal-Sprayed Coatings. Master’s Thesis.

[146] Zhou J., Zeng Q., Xiong Y., Xu J., Zhang F., Wang D., Zheng J. (2022). Research on the shielding performance and optimization of new type foam metal matrix composite shielding materials. Nucl. Instrum. Methods Phys. Res. Sect. B Beam Interact. Mater. At..

[147] Zhang P., Xu W.R., Wang W.X., Li J., Jia C.P., Ma Y.F. (2020). Preparation Method of Gadolinium Oxide/Tungsten/Aluminum Neutron and Gamma-Ray Core–Shell co-Shielding Material. Chinese Patent.

[148] Demir İ., Gümüş M., Gökçe H.S. (2020). Gamma ray and neutron shielding characteristics of polypropylene fiber-reinforced heavyweight concrete exposed to high temperatures. Constr. Build. Mater..

[149] Nikbin I.M., Mohebbi R., Dezhampanah S., Mehdipour S., Mohammadi R., Nejat T. (2019). Gamma ray shielding properties of heavy-weight concrete containing nano-TiO_2_. Radiat. Phys. Chem..

[150] Al-Tersawy S.H., El-Sadany R.A., Sallam H.E.M. (2021). Long-term behavior of normal weight concrete containing hybrid nanoparticles subjected to gamma radiation. Arch. Civ. Mech. Eng..

[151] Abo-El-Enein S.A., El-Hosiny F.I., El-Gamal S.M.A., Amin M.S., Ramadan M. (2018). Gamma radiation shielding, fire resistance and physicochemical characteristics of Portland cement pastes modified with synthesized Fe_2_O_3_ and ZnO nanoparticles. Constr. Build. Mater..

[152] Mokhtari K., Kheradmand Saadi M., Ahmadpanahi H., Jahanfarnia G. (2021). Fabrication, characterization, simulation and experimental studies of the ordinary concrete reinforced with micro and nano lead oxide particles against gamma radiation. Nucl. Eng. Technol..

[153] Dezhampanah S., Nikbin I.M., Mehdipour S., Mohebbi R., Moghadam H. (2021). Fiber-reinforced concrete containing nano-TiO_2_ as a new gamma-ray radiation shielding materials. J. Build. Eng..

[154] Tekin H.O., Sayyed M.I., Issa S.A.M. (2018). Gamma radiation shielding properties of the hematite–serpentine concrete blended with WO_3_ and Bi_2_O_3_ micro and nano particles using MCNPX code. Radiat. Phys. Chem..

[155] Tekin H.O., Singh V.P., Manici T. (2017). Effects of micro-sized and nano-sized WO_3_ on mass attenuation coefficients of concrete by using MCNPX code. Appl. Radiat. Isot..

[156] El-Nahal M.A., Elsafi M., Sayyed M.I., Khandaker M.U., Osman H., Elesawy B.H., Saleh I.H., Abbas M.I. (2021). Understanding the effect of introducing micro- and nanoparticle bismuth oxide (Bi_2_O_3_) on the gamma ray shielding performance of novel concrete. Materials.

[157] Hassanzadeh M., Sadat Kiai S.M. (2018). Calculation of photon attenuation coefficient and dose rate in concrete with the addition of SiO_2_ and MnFe_2_O_4_ nanoparticles using MCNPX code and comparison with experimental results. Nucl. Sci. Tech..

[158] Florez R., Colorado H.A., Alajo A.B., Giraldo C.H.C. (2019). The material characterization and gamma attenuation properties of Portland cement–Fe_3_O_4_ composites for potential dry cask applications. Prog. Nucl. Energy.

[159] Nikbin I.M., Shad M., Jafarzadeh G.A., Dezhampanah S. (2019). An experimental investigation on combined effects of nano-WO_3_ and nano-Bi_2_O_3_ on the radiation shielding properties of magnetite concretes. Prog. Nucl. Energy.

[160] Mesbahi A., Ghiasi H. (2018). Shielding properties of the ordinary concrete loaded with micro- and nano-particles against neutron and gamma radiations. Appl. Radiat. Isot..

[161] Özdemir H.G., Kaçal M.R., Akman F., Polat H., Agar O. (2023). Investigation of gamma radiation shielding characteristics of bismuth reinforced ternary composites in wide photon energy region. Radiat. Phys. Chem..

[162] Chen Z.F., Xiao L.F., Tao Q.W., Xie L.P. (2019). Research on the effect of lead–zinc tailings sand on the gamma-ray shielding performance of concrete. Ind. Constr..

[163] Heerasingh M., Sankarappa T., Malge A., Devidas A., Raghavendra B., Pallavi J., Dyama A. (2023). Dielectric, thermal and gamma shielding characteristics of PbO–TeO_2_–V_2_O_5_–CoO glasses. Mater. Chem. Phys..

[164] Marzuki A., Sasmi T., Fausta D.E., Harjana H., Suryanti V., Kabalci I. (2023). The effect of Bi_2_O_3_/PbO substitution on physical, optical, structural, and gamma shielding properties of boro-tellurite glasses. Radiat. Phys. Chem..

[165] Ardiansyah A., Heryanto H., Armynah B., Salah H., Sulieman A., Bradley D.A., Tahir D. (2023). Physical, mechanical, optical, and gamma radiation shielding properties of the BaO-based glass system prepared by the melt-quench technique: A review. Radiat. Phys. Chem..

[166] Sayyed M.I., Mhareb M., Şakar B.C., Mahmoud K., Şakar E., Thabit H.A., Kaky K.M., Baki S. (2024). Experimental investigation of structural and radiation shielding features of Li_2_O-BaO-ZnO-B_2_O_3_-Bi_2_O_3_ glass systems. Radiat. Phys. Chem..

[167] Sayyed M.I., Alrashedi M.F., Almuqrin A.H., Elsafi M. (2022). Recycling and optimizing waste lab glass with Bi_2_O_3_ nanoparticles to use as a transparent shield for photons. J. Mater. Res. Technol..

[168] Cheewasukhanont W., Limkitjaroenporn P., Kothan S., Kedkaew C., Kaewkhao J. (2020). The effect of particle size on radiation shielding properties for bismuth borosilicate glass. Radiat. Phys. Chem..

[169] Saleh E.E., Algradee M.A., El-Fiki S.A., Youssef G.M. (2022). Fabrication of novel lithium lead bismuth borate glasses for nuclear radiation shielding. Radiat. Phys. Chem..

[170] Mhareb M.H.A., Ghrib T., Sayyed M., Hamad M.K., Sfina N., Ben Ali A., Almessiere M. (2024). Structural, morphological, optical, and radiation shielding properties for BaO–TeO_2_ glass ceramic modified with different oxides: Bi_2_O_3_, MoO_3_, MnO_2_, and TiO_2_. Phys. B Condens. Matter.

[171] Shah A.Z., Zaid M.H.M., Matori K.A., Yaakob Y., Sarmani A.R., Hisam R. (2024). Comprehensive study on structural, elastic and radiation shielding abilities of novel quaternary Bi_2_O_3_–TeO_2_–Li_2_O–Al_2_O_3_ glasses. Prog. Nucl. Energy.

[172] Mhareb M.H.A., Alajerami Y.S.M., Sayyed M.I., Mahmoud K.A., Ghrib T., Hamad M.K., Drmosh Q.A., Sfina N., Almessiere M.A. (2022). Morphological, optical, structural, mechanical, and radiation-shielding properties of borosilicate glass–ceramic system. Ceram. Int..

[173] Issa S.A.M., Kassab L.R.P., Susoy G., Nishimura M.V.M., da Silva Mattos G.R., Bordon C.D.S., Tekin H.O. (2020). Fabrication, optical characteristic, and nuclear radiation shielding properties of newly synthesised PbO–GeO_2_ glasses. Appl. Phys. A.

[174] Prabhu N.S., Hegde V., Sayyed M.I., Agar O., Kamath S.D. (2019). Investigations on structural and radiation shielding properties of Er^3+^ doped zinc bismuth borate glasses. Mater. Chem. Phys..

[175] El-Sharkawy R.M., Shaaban K.S., Elsaman R., Allam E.A., El-Taher A., Mahmoud M.E. (2020). Investigation of mechanical and radiation shielding characteristics of novel glass systems with the composition xNiO–20ZnO–60B_2_O_3_–(20–x)CdO based on nanometal oxides. J. Non-Cryst..

[176] Sadeq M., Bashter I., Salem S., Mansour S., Saudi H., Sayyed M., Mostafa A. (2022). Enhancing the gamma-ray attenuation parameters of mixed bismuth/barium borosilicate glasses: Using an experimental method, Geant4 code and XCOM software. Prog. Nucl. Energy.

[177] Rao L.S., Hila F.C., Reddy M.S., Hussain S. (2024). Effect of zirconium oxide nanoparticles on thermal, optical, and radiation shielding properties of Bi_2_O_3_–B_2_O_3_–MnO_2_ glasses. Appl. Radiat. Isot..

[178] Selim Y., Sallam O.I., El-Alaily N.A. (2024). Investigation of some optical, conducting and radiation shielding properties of Dy^3+^ ions doped boro-silicate glass systems. Ceram. Int..

[179] Alzahrani J.S., Sharma A., Nazrin S.N., Alrowaili Z.A., Al-Buriahi M.S. (2022). Optical and radiation shielding effectiveness of a newly fabricated WO_3_ doped TeO_2_–B_2_O_3_ glass system. Radiat. Phys. Chem..

[180] Kavgacı M., Yaykaşlı H., Eskalen H., Kavun Y., Yaşar M.M. (2024). BaTiO_3_–B_2_O_3_–MgO–Na_2_O–CaO glass series: Radiation shielding, thermal, and mechanical properties. Nucl. Instrum. Methods Phys. Res. Sect. B Beam Interact. Mater. At..

[181] Çağlar M., Karabul Y., Kılıç M., Özdemir Z.G., İçelli O. (2021). Na_2_Si_3_O7/Ag micro and nano-structured glassy composites: The experimental and MCNP simulation surveys of their radiation shielding performances. Prog. Nucl. Energy.

[182] Elsafi M., El-Nahal M.A., Sayyed M.I., Saleh I.H., Abbas M.I. (2021). Effect of bulk and nanoparticle Bi_2_O_3_ on attenuation capability of radiation shielding glass. Ceram. Int..

[183] Elias J.A., Montes E., Torres-Castro A., Wiechers C., Gomez-Solis C., Vega-Carrillo H.R., Sosa M.A., Vallejo M. (2022). Mn, Cu and Cr nanoparticles in Li_2_B_4_O_7_ glass: Radiation shielding and optical properties. Radiat. Phys. Chem..

[184] Mahmoud M.E., El-Khatib A.M., Halbas A.M., El-Sharkawy R.M. (2020). Investigation of physical, mechanical and gamma-ray shielding properties using ceramic tiles incorporated with powdered lead oxide. Ceram. Int..

[185] Mahmoud M.E., El-Khatib A.M., Halbas A.M., El-Sharkawy R.M. (2021). Ceramic tiles doped with lead oxide nanoparticles: Their fabrication, physical, mechanical characteristics and γ-ray shielding performance. Radiat. Phys. Chem..

[186] Asal S., Erenturk S.A., Haciyakupoglu S. (2021). Bentonite based ceramic materials from a perspective of gamma-ray shielding: Preparation, characterization and performance evaluation. Nucl. Eng. Technol..

